# Endothelial cell elongation and alignment in response to shear stress requires acetylation of microtubules

**DOI:** 10.3389/fphys.2024.1425620

**Published:** 2024-09-10

**Authors:** Katiannah Moise, Keerthana M. Arun, Maalavika Pillai, Jocelynda Salvador, Aarya S. Mehta, Yogesh Goyal, M. Luisa Iruela-Arispe

**Affiliations:** ^1^ Department of Cell and Development Biology, Northwestern University, Feinberg School of Medicine, Chicago, IL, United States; ^2^ Center for Synthetic Biology, Northwestern University, Chicago, IL, United States

**Keywords:** cytoskeleton, hemodynamics, mechanotransduction, mechanosensing, vascular biology

## Abstract

The innermost layer of the vessel wall is constantly subjected to recurring and relenting mechanical forces by virtue of their direct contact with blood flow. Endothelial cells of the vessel are exposed to distension, pressure, and shear stress; adaptation to these hemodynamic forces requires significant remodeling of the cytoskeleton which includes changes in actin, intermediate filaments, and microtubules. While much is known about the effect of shear stress on the endothelial actin cytoskeleton; the impact of hemodynamic forces on the microtubule network has not been investigated in depth. Here we used imaging techniques and protein expression analysis to characterize how pharmacological and genetic perturbations of microtubule properties alter endothelial responses to laminar shear stress. Our findings revealed that pharmacological suppression of microtubule dynamics blocked two typical responses to laminar shear stress: endothelial elongation and alignment. The findings demonstrate the essential contribution of the microtubule network to changes in cell shape driven by mechanical forces. Furthermore, we observed a flow-dependent increase in microtubule acetylation that occurred early in the process of cell elongation. Pharmacological manipulation of microtubule acetylation showed a direct and causal relationship between acetylation and endothelial elongation. Finally, genetic inactivation of aTAT1, a microtubule acetylase, led to significant loss of acetylation as well as inhibition of cell elongation in response to flow. In contrast, loss of HDAC6, a microtubule deacetylase, resulted in robust microtubule acetylation with cells displaying faster kinetics of elongation and alignment. Taken together, our findings uncovered the critical contributions of HDAC6 and aTAT1, that through their roles in the regulation of microtubule acetylation, are key mediators of endothelial mechanotransduction.

## Introduction

The inner surface of the entire circulatory system is covered by a monolayer of endothelial cells (EC) directly exposed to the physical stressors of blood flow, namely: stretch, pressure, and shear stress (Chien, 2007; Baeyens and Schwartz, 2016). In response to shear stress, endothelial cells elongate in the direction of flow and transduce mechanical stimuli into sequential biochemical reactions that enable homeostatic regulation of vascular function (Bevan et al., 1990; Langille, 1992). In addition, endothelial cells significantly alter their transcriptome and epigenome affecting their inflammatory status. It has been very well documented that the type of flow, laminar or disturbed, can have profound effects on endothelial gene expression (Chiu and Chien, 2011). In this manner, regions associated with non-uniform shear stresses like areas of bifurcations induce a pro-inflammatory status on the endothelium and are prone to the development of early atherosclerotic lesions. In contrast, regions of the vessel wall exposed to uniform laminar flow are anti-inflammatory and athero-protective.

The realization that physical forces regulate the inflammatory status of the endothelium by affecting gene expression stimulated interest in clarifying the mechanisms associated with mechanosensing and mechanotransduction. To identify the molecular players involved in the cellular responses to mechanical stress, endothelial cells have been cultured under flow-controlled conditions and subjected to uniform, disturbed, pulsatile and oscillatory types of shear stresses to mimic *in vivo* environments. In this manner, a large array of mechanosensing receptors, ion channels, and cell adhesion molecules have been identified as playing important roles in flow responses and their respective contributions have been studied in depth (Coste et al., 2010; Mack et al., 2017; Fleming et al., 2005; Mendoza et al., 2010; Mohan et al., 1997; Zhang et al., 2006; Tzima et al., 2005; Fan et al., 2017). More recently, the potential impact of shear stress patterns in the development of site-specific vascular malformations added further attention to the cross talk between physical forces and signal transduction in the emergence of vascular pathology (Rodel et al., 2019).

Perhaps the most well recognized effect of laminar shear stress relates to its impact on endothelial cell shape (Campinho et al., 2020). Confluent endothelial monolayers subjected to physiological levels of laminar shear stress elongate in direction to flow within 24 h. The transition in cell shape from polygonal to elongated implies significant remodeling of the cytoskeleton contingent upon exposure to flow. Measurements obtained using live cell microscopy revealed that shear stress induces heterogenous patterns of mechanical strain on the intermediate filament network at the basal surface of endothelial cells, and that this affects focal adhesions (Helmke et al., 2003). Lateral strain caused by shear stress on PECAM homotypic interactions was shown to impact vimentin and actin. These collective dynamic structural changes can also concentrate mechanical stimuli in cellular compartments where biochemical transduction occurs. For example, mean traction force against the substrate and Rho GTPase activity were found to be increased in endothelial cells shortly after onset of shear stress (Shiu et al., 2004; Collins et al., 2012). Furthermore, activation of Rho followed by Rac triggered robust cellular changes in the actin cytoskeleton with the formation of stress fibers and reorganization of focal adhesions (Mott and Helmke, 2007). Finally, the microtubule (MT) network is also affected by shear stress. Using subconfluent endothelial cells, Zielinski and colleagues showed a rapid reorganization of microtubules upon application of shear stress (Zielinski et al., 2018). Importantly, laminar shear stress was also shown to promote microtubule stability by increasing acetylation downstream of GSK-3beta (McCue et al., 2006). This last study indicated that microtubules were altered in response to shear stress, but the biological impact of this alteration on individual and collective endothelial cell responses has not been systematically examined.

In this study, we sought to investigate the requirement of the MT network to endothelial cells responses to shear stress and gain additional molecular insight on mechanisms. We found that MT dynamics were essential to enable elongation and alignment following application of laminar shear stress on endothelial monolayers. We confirmed that shear stress promotes rapid and robust microtubule acetylation and that this effect was sustained over 48 h *in vitro*. We demonstrated that MT acetylation is higher in areas of laminar shear than in regions of disturbed flow *in vivo*. We further showed that flow-mediated increase in acetylation is driven by a rapid decrease in HDAC6 protein. Finally, we demonstrated that pharmacological or genetic perturbations in microtubule acetylation blocked the ability of endothelial cells to elongate and align in response to laminar shear stress.

## Materials and methods

### Cell culture

Human arterial endothelial cells (HAECs) were purchased from the American Type Culture Collection (ATCC, #PCS-100-011, Manassas, VA) and Lonza (Lonza, #CC-2535, Morrisville, NC). HAECs were cultured on 0.1% gelatin coated dishes and used between passages 4 and 8. Human Umbilical Vein Endothelial cells (HUVEC) were isolated from three separate patients and used between passages 4 and 9. Patients were informed and gave their consent for the research. Both HAECs and HUVECs were cultured in commercial MCDB-131 media (MCDB131-WOFBS, VEC Technologies, Inc.) or in commercial EBM -2 Basal media (Lonza, #CC-3121) supplemented with EGM-2 BulletKit (Lonza, #CC-4176) containing 10% fetal bovine serum (FBS; Omega Scientific Inc.) with antibiotics. Cultures were maintained in a 5% CO_2_ incubator at 37°C. Human embryonic kidney cells (HEK293T) (ATCC, #CRL-3216) were cultured with DMEM media with 10% FBS (Fisher Scientific #MT10017CV). All cells were characterized with cell-specific antibodies and tested negative for *mycoplasma*.

### *In vitro* simulation of laminar shear stress

#### Orbital shaker method

Confluent endothelial cells (HAEC and HUVEC) were cultured in gelatin-coated 6-well glass bottom plates with 4% Dextran media with 1% or 10% FBS and subjected to constant fluid shear stress (20 dynes/cm^2^) on a Benchmark Orbi-Shaker Jr Mini orbital shaker (130 rpm, 19 mm radius, Sycamore Life Sciences) inside an incubator with 5% CO_2_ and at 37°C incubator. Dextran (Leuconostoc spp. Mr 450,000-650,000, Millipore Sigma, #31392) was added to the media at a w/v of 4% to create a viscosity similar to blood and delivered the adequate shear stress.

#### Ibidi pump system method

For some experiments, we also used an Ibidi pump system (Ibidi #10902) to deliver laminar shear stress at 20 dynes/cm^2^. For this, 100,000 endothelial cells were seeded into a 0.4 mm deep u-slide I Luer (Ibidi #80176) microfluidic slide 24 h prior to experiments. A media change was performed prior to connecting the slide to the fluidic unit.

### Microtubule targeting agents (MTAs)

Taxol (10 nM, Tocris #1097), Nocodazole (100 nM, Selleck Chemicals #S2775), and Colchicine (10 nM, Selleck Chemicals #S2284) were applied to endothelial monolayers either under static or flow conditions, as indicated. The concentrations were selected by using EB3-GFP transfections. The lowest concentration able to achieve microtubule disturbance in the absence of cell death was selected.

### Pharmacological perturbation of acetylation

Confluent HAECs seeded on glass bottom plates were treated with the TAK1 inhibitor, 5Z-7-Oxozeaenol (5Z-7; 1uM; Fisher Scientific #HY-12686) to suppress acetylation mediated by alpha-TAT1 (Shah et al., 2018). Tubacin (Tub; 5uM; Tocris #3402) was used to inhibit HDAC6 activity. DMSO was used as a control.

### Immunofluorescence

To visualize microtubules, post-translational modifications of microtubules, and cell-cell junctions, two methods of fixation and staining were employed. Endothelial cultures were washed with 1x PBS buffer and fixed with ice-cold methanol for 10 min at −20°C to visualize microtubules. Wells were permeabilized with 0.1% Triton X-100 in 1x PBS buffer for 10 min at room temperature. Depending on the fixation, methanol-fixed cells were stained using monoclonal rabbit Acetylated -α-tubulin (Lys40) (D20G3) (Cell Signaling #5335, RRID:AB_10544694, 1:1,000), monoclonal mouse anti-Acetylated α-tubulin (Millipore Sigma #T7451, RRID:AB_609894, 1:1,000), monoclonal mouse anti-Acetylated (Lys40) (6-11B-1) α-tubulin (Cell Signaling #12152s, RRID:AB_2797830, 1:1,000), monoclonal mouse DM1a (Cell Signaling #3873s, RRID:AB_1904178, 1:1,000), polyclonal rabbit anti-detyrosinated tubulin (Millipore Sigma #AB3201, RRID:AB_117350 1:1,000), monoclonal mouse anti-polyglutamylated Tubulin, clone B3 (Sigma-Aldrich #T9822,RRID:AB_477598, 1:1,000), monoclonal rat Anti-Tubulin Antibody, clone YL1/2 (Tyrosinated-tubulin) (Millipore Sigma #MAB1864, RRID:AB_1679330, 1:1,000) and DAPI for nuclear staining (4′, 6-Diamidino-2-Phenylindole, Dihydrochloride) (Thermo Fisher Scientific, #D1306, RRID:AB_2629482, 5 mg/mL). Primary antibodies were incubated for 1 h at room temperature. Cell-cell junctions were visualized using a 647-conjugated Hec1/VE-cadherin supernatant gifted by William Muller (Northwestern University, Chicago, IL, 1:500) for 30 min at room temperature. Conjugation of the antibody was done using a Pierce Dylight Antibody labeling Kit (Fisher Scientific #PI84535). For time-lapse visualization of microtubules under laminar flow, a red live dye was used by ABBERIOR (2.5 uM, #LVRED-0141050UG). Secondary antibodies were used at 1:400 concentration for Donkey anti-mouse IgG (H&L) (Alexa Fluor 568) (Abcam, #ab175472, RRID:AB_2636996), Donkey anti-mouse IgG (H&L) (Alexa Fluor 488) (Abcam, #b150105; RRID:AB_2732856), Donkey anti-rabbit IgG (H&L) Alexa Fluor 568 (Thermo Fisher Scientific, #A10042; RRID:AB_2534017) and incubated for 1 h at room temperature. For methanol fixed samples, all antibodies were diluted in Wash Buffer Solution (1% BSA, 10% of 1x TBS, 0.1% of Triton X-100 in ddH20) and all washes were done using the Wash Buffer solution.

For PFA-fixed endothelial cultures, cells were stained using DAPI and polyclonal goat human VE-Cadherin (R&D Systems, #AF938, 1:500) as primary antibodies with overnight incubation at 4°C. For secondary antibodies, all were used at 1:400 dilution using Donkey-anti-goat IgG Alexa 647 pre-adsorbed (Abcam, #ab150135; RRID:AB_2687955) Secondaries were incubated for 2 h at room temperature. Antibodies were diluted in a buffer solution containing 3% normal donkey serum (Jackson ImmunoResearch), 0.3% Triton X-100 (Sigma-Aldrich), 0.05% Tween20 (Sigma-Aldrich) in 1xPBS. Washes were done with 1x PBS. Immunostaining of whole-mount aorta was performed as previously described (McDonald et al., 2018).

Images were taken at ×20 magnification using an Olympus PlanApochromat 20x/0.8 N.A. objective on the ECHO Revolve scope (ECHO, San Diego, CA). Other images were taken at ×68 magnification using a CFI60 PlanApochromat Lambda D 40x/1.25 N.A. silicone oil objective on a Nikon Eclipse Ti2 spinning disk confocal microscope with CSU-W1 Yokogawa Confocal Scanner unit with LIVESR for acquisition. Images taken on the Nikon confocal microscope were post-processed using the deconvolution feature with the Richardson-Lucy method. Additional image processing and quantification was done using Imaris Software 9.9 and 10.0 (Bitplane).

### Immunoblotting

Cultures were washed twice with 1xPBS buffer and protein extracts were collected with cell scrapers in mRIPA lysis buffer (40 mM Tris-HCL pH 7.5, 10% glycerol, 5mM MgCl_2_, 4%SDS), containing phosphatase and protease inhibitors. Lysates were spun down and the supernatant was removed for determination of protein concentration (using a Bradford Assay) and storage. For Western blots, equal concentrations of protein lysates were loaded onto the wells of 4%–15% Mini-Protean TGXTM gels (BioRad #4561096) or 4%–20% Criterion TGX Stain-Free Protein Gels (BioRad #5678093) and subjected to electrophoresis under reducing conditions. Gels were transferred onto nitrocellulose membranes (BioRad Laboratories) and blocked for 1 h at room temperature with 2% dry skim milk-1xTBS buffer with 0.1% Tween-20. Primary antibodies monoclonal rabbit Acetylated -α-tubulin (Lys40) (D20G3) (Cell Signaling #5335, RRID:AB_10544694, 1:1,000), monoclonal mouse anti-Acetylated α-tubulin (Millipore Sigma #T7451, RRID:AB_609894, 1:1,000), monoclonal mouse anti-Acetylated (Lys40) (6-11B-1) α-tubulin (Cell Signaling #12152s, RRID:AB_2797830, 1:1,000), monoclonal mouse DM1a (Cell Signaling #3873s, RRID:AB_1904178, 1:1,000), polyclonal rabbit anti-detyrosinated tubulin (Millipore Sigma #AB3201, RRID:AB_117350 1:1,000), monoclonal mouse anti-polyglutamylated tubulin, clone B3 (Sigma-Aldrich #T9822,RRID:AB_477598, 1:1,000), monoclonal rat Anti-Tubulin Antibody, clone YL1/2 (Tyrosinated-tubulin) (Millipore Sigma #MAB1864, RRID:AB_1679330, 1:1,000), mouse monoclonal GAPDH (Millipore Sigma #MAB374, RRID:AB_2107445, 1:1,000), monoclonal rat α-tubulin (YOL1/34) (Invitrogen #MA1-80189, RRID:AB_2210200, 1:1,000) and monoclonal rabbit HDAC6 (D2E5) antibody (Cell Signaling #7558s, RRID:AB_10891804, 1:1,000) were used. Blots were incubated overnight at 4°C with primaries. Membranes were washed with 1xTBS with Tween-20 buffer before adding secondary horseradish peroxidase-conjugated anti-rabbit, anti-mouse or anti-rat antibodies. Visualization of bands was done using enhanced luminol-based chemiluminescence (SuperSignal West Femto Maximum Sensitivity Substrate) (Fisher Scientific #P134096) or (SuperSignal West Pico Chemiluminescent Substrate) (Fisher Scientific #PI34580). Densitometry was performed to quantify protein amount using Image Lab (Bio rad). Normalization of protein levels was performed against housekeeping genes: GAPDH or α-tubulin.

### Generation of HDAC6 and aTAT1 CRISPR-KO endothelial cells

HUVECs between a passage of 4 and 7 were seeded in antibiotic-free culture media with 10% FBS with a final concentration of 8ug/mL of polybrene and MOI 10 of lentivirus. Constructs containing CAS9 and guide RNAs (as in Table 1) were packaged with a second generation system using 0.08 mg/mL of PEI, VSV. G, psPAX2, and transfer plasmids in OPTIMEM I reduced serum medium. For generation of virus, transfection was performed by adding dropwise the mixed solution onto a 15 cm plastic tissue culture dish of HEK293Ts at 90% confluency cultured in antibiotic free DMEM media with 10% FBS. Lentiviral supernatants were collected 1 day after transfection. Transduced HUVECs were selected for positive genetic inactivation by puromycin selection for 2 weeks.

**TABLE 1:** Lentiviral constructs.

Nickname	Construct	Guide sequences	Length of region of deletion	Vector size
HDAC6KO	pLV[2CRISPR]hCas9:T2A:Puro-U6>hHDAC6 [gRNA#1780]-U6>hHDAC6[gRNA#2152]	TCC​ATC​CAC​CGC​TAC​GAG​CA, ATG​ATC​CGC​AAG​ATG​CGC​TG	*1,693*	*12,124 bp*
αTAT1KO	pLV[2CRISPR]hCas9:T2A:Puro-U6>hATAT1 [gRNA#170]-U6>hATAT1[gRNA#221]	GAGCATGTGGCACTCACGGT, CATGAGTCTGTGCAACGCCA	378	12,047* *bp

### Transfection of EB3-GFP plasmid

Transfections of endothelial cells were performed using a Lipofectamine 3000 kit (Thermo Fisher Scientific # L3000015). EB3-GFP plasmid (Addgene, Plasmid #190164), Lipofectamine 3000 Reagent and P3000 Reagent were diluted in Opti-MEM I reduced serum medium (Fisher Scientific # 31985070) combined, and incubated at room temperature for 10–15 min. In a 6-well plastic bottom plate, 1.75 mL of antibiotic-free media with 3% FBS were added to each well. Confluent cells were trypsinized (Thermo Fisher Scientific Cat#15090046) and neutralized with full serum media, spun down, and counted for a cell concentration of 200,000 cells/mL. Incubated transfection reagent was added to each well, after which, the appropriate volume of cells was added to plates. The next day, a media change was performed using EGM-2 media with 10% FBS and antibiotics.

### Time-lapse live-imaging of EB3-GFP transfected endothelial cells

24 h prior to live-imaging, 120,000 transfected endothelial cells are seeded into 0.4 mm deep u-slide I Luer microfluidic slides. Cells were used between 1-2 days after transfection to ensure the best transfection efficiency. Cells within the chamber are allowed to attach overnight. A Nikon Eclipse Ti2 spinning disk confocal microscope was used for live-imaging. A temperature-controlled enclosure with a heated stage tabletop incubator (Ibidi, Cat# 12720) was used. The attached u-slide is placed into the stage top incubator at 35°C for the lid and 37°C for the stage. To follow EB3 comets, cells were visualized using the Perfect Focus feature. Using the ND multipoint acquisition, multiple cells were chosen to be imaged simultaneously. Images were taken at intervals of 2s for a duration of 2 min with 200 ms exposure time and a 15% power of the 488 nm-laser line. Once the time-lapse videos were acquired, movies were post-processed using denoise (Denoise.ai) on NIS Elements for all frames.

### Elongation factor and alignment analysis

All images were segmented using the “cyto2” model in CellPose 2.0, a pre-trained neural network-based cell segmentation algorithm. CellPose (Stringer et al., 2021) uses a U-net architecture to estimate spatial flow, combined with an energy function for each mask (i.e. the region covered by the outlines of a cell). Although CellPose is pre-trained on many diverse datasets, it is a semi-supervised model and requires certain user-defined parameters for ensuring accurate segmentation. Three main parameters that can be modified in this model are the average cell diameter, flow, and cell probability threshold. The average cell diameter (in pixels) can either be user defined or algorithmically estimated. The flow is defined as the model fit threshold, and it determines whether a mask is consistent with the real cell’s signal. Increasing the flow generates more masks. The cell probability threshold determines whether a region is a cell based on the channel intensity. By setting a high cell probability threshold, dim areas of the image can be detected as masks. Conversely, setting a low probability threshold avoids detection of dim areas as masks. Various properties of the mask (such as area, orientation, major axis length, etc.) are identified using the region_props function in the scikit-image package, which is commonly used to measure region properties of labeled images. Following segmentation, the masks were quality-checked to avoid false-positive segmentations. Very small segmentations (less than 20% of the largest cell), oddly shaped segments (major to minor axis ratio greater than 10) and partial cells present on the border of the image were removed during this process. The masks were used to estimate the longest diameter, which is the major axis of the cell. The chord perpendicular to the major axis and passing through the centroid of the mask is called the minor axis of the cell. The elongation factor is calculated as the ratio of the major axis to the minor axis. The orientation is estimated as the angle of the major axis from a horizontal line (*x*-axis). Since cells can be oriented along any axis (not necessarily the *x*-axis), we subtracted each value from the median orientation of cells in that image, to obtain the deviation from the median value (which is transformed to 0). The cell orientations are then represented as a diameter in a cell using a radial plot.

### Illustrations

Illustrations were made with Biorender.

### Statistical analysis

Statistical analyses across conditions were performed using GraphPad Prism 9 (San Diego, United States). For normally distributed data, two-tailed unpaired t-test was used to determine statistically significant differences between two groups. For nonparametric data, a two-tailed Mann-Whitney U test was conducted. For groups with three or more conditions, Kruskal-Wallis test was performed. For Western blot analyses, non-normal data was performed using a Wilcoxon sign rank test, while normally distributed data utilized a one sample t-test. All samples were done with at least 3 technical replicates, identified biological replicates are disclosed within each individual figure legend. *p* < 0.05 was considered statistically significant for all analyses.

## Results

### Active microtubule remodeling is required for EC elongation and alignment in response to laminar shear stress

**FIGURE 1:**
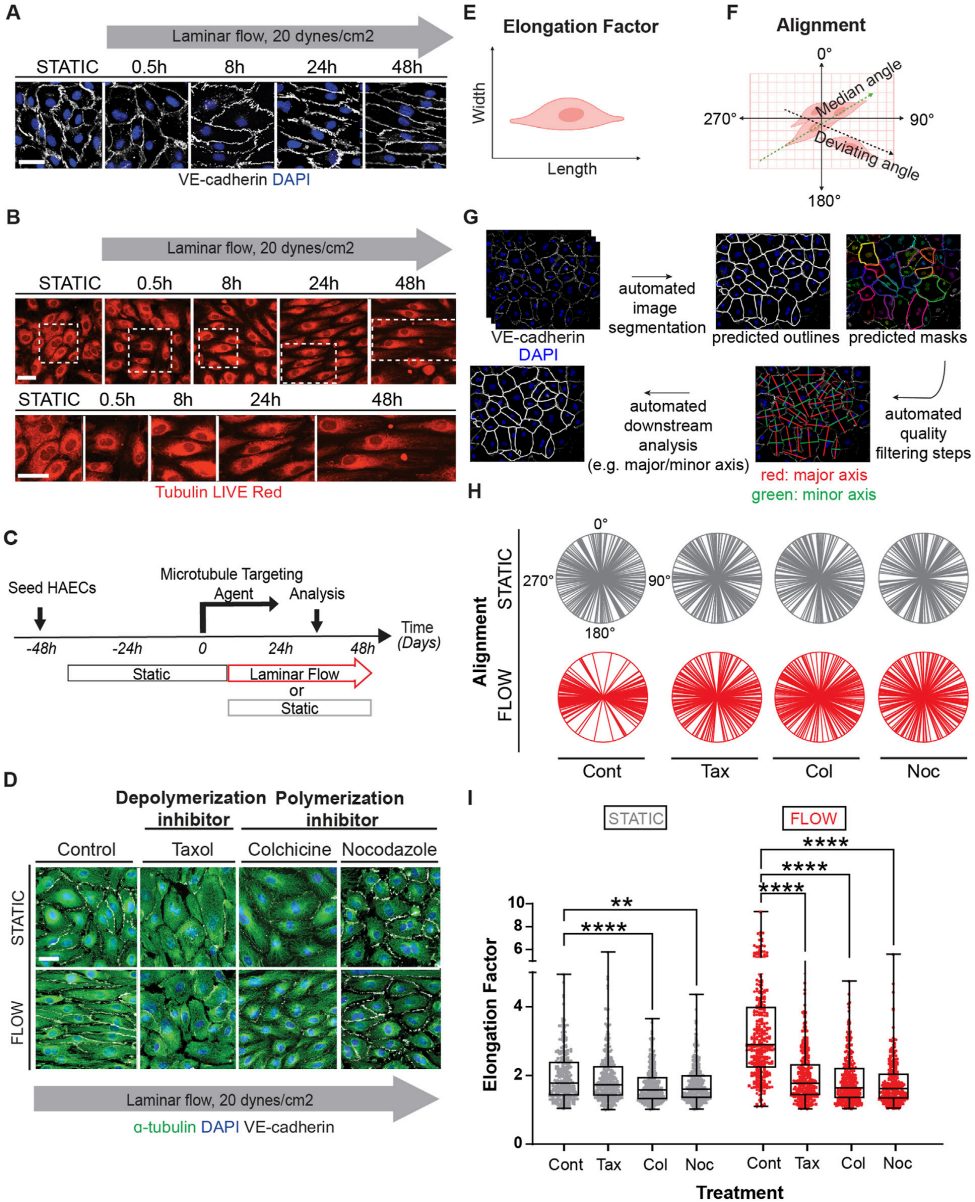
Microtubule dynamics are required for proper endothelial cell responses to shear stress. Human aortic endothelial cell (HAEC) monolayers shown at resting conditions (static) or exposed to laminar flow at various time points: 0.5, 8, 24, 48 h were assessed for cell shape and alignment. **(A)** Cells under flow were stained for VE-cadherin (white) and nuclei (DAPI). Grey arrow indicates the direction and velocity of laminar flow. Scale bar: 40 um. **(B)** Live red dye that binds to tubulin shows microtubule reorganization. Scale: 20 um. **(C)** Confluent endothelial cells treated with microtubule targeting agents (MTAs); taxol, colchicine and nocodazole or DMSO (control) before exposure to flow or still conditions. **(D)** Immunofluorescence staining of microtubules (green) in control and MTA-treated cells in the presence and absence of shear stress. Cells were stained for nuclei (DAPI), and VE-cadherin (white). Scale bar: 20 um. **(E)** Elongation Factor: cell shape as analyzed by comparing the length over the width of a cell. **(F)** Alignment: defined by the relative positioning of a cell to the median angle of a monolayer of cells. **(G)** Cell shape analysis was performed by segmenting images using the “cyto2” model in CellPose 2.0, a pre-trained neural network-based algorithm, to quantify cell dimensions **(H)** Radial graphs comparing control and MTA treated cells. Values are shown between 0 to 360 degrees. *n* = 100 cells per condition, 3 biological replicates. Coefficients of variation for static (left to right) are 0.5, 0.56, 0.51, 0.6 and for flow (left to right) are 0.29, 0.5, 0.52, and 0.56 (Supplementary Figure S2). **(I)** Quantification of elongation factor. *n* = 300 per condition, 3 biological replicates. Values are shown as mean ± SD; *n* = 300 cells per condition; Kruskal-Wallis Test with Dunn’s correction.*p < 0.05, **p < 0.01, ***p < 0.001, ****p < 0.0001.

In response to laminar shear stress, endothelial cells progressively alter their shape, transitioning from polygonal to elongated morphology, and aligning in the direction of flow (Figure 1A). This process is gradual but noticeable by 24–48 h in confluent monolayers. Importantly, exposure of subconfluent cells to laminar shear stress, show that cell-cell contacts were not necessary for elongation but were required for alignment (orientation of cells in relation to each other) (Supplementary Figure S1A–C). Changes in elongation and alignment are the most basic responses to shear stress and are known to be associated with changes in all three major elements of the cytoskeleton (Zielinski et al., 2018). Significant emphasis has been placed on understanding the effects of flow dynamics on actin, while information associated with changes in the microtubular network has lagged. To capture information associated with microtubule remodeling, we first used sirTubulin to facilitate live cell labeling of MTs. Using this approach, we observed changes in orientation and structure of MTs that indicated a complex and progressive response following exposure of endothelial monolayers to laminar flow (Figure 1B). Still images collected at specific time points revealed rapid expansion of the MT network as cells elongate and increase in the abundance of polymerized MTs (Figure 1B). Importantly, under subconfluent conditions, elongation was noted, but not alignment (Supplementary Figure S1A–C), indicating that cell-cell interactions were necessary for alignment.

To clarify the significance of the flow induced MT dynamics, we exposed endothelial cultures to well-known pharmacological drugs that either promote (colchicine, nocodazole) or prevent (taxol) depolymerization of MTs (Figure 1C). MT-disruptive agents (MTAs) were titrated using EB3-GFP transfections (Supplementary Figure S1D, Video S1). The lowest dose that affected EB3-GFP movement was selected to evaluate their effect on elongation. Interestingly all MT-disrupting agents blocked the ability of endothelial cells to respond to laminar shear stress (Figure 1D). The effects on elongation (length over width) and alignment (mean angle of cell orientation in relation to each other) (Figures 1E, F) were quantified on confluent monolayers using several biological replicates and endothelial cell subtypes (aortic and vein) by combining machine learning and artificial intelligence (AI) strategies to process hundreds of individual images (Figure 1G). Radial plots were used to represent cell alignment, whereby polarity in two directions indicated positive alignment and broad circular distribution represented lack of alignment. This robust assembly of quantification methods indicated that all three pharmacological treatments disrupted endothelial cell alignment and elongation under confluent conditions (Figures 1H, I).

Figure 1I includes findings from three biological replicates from each treatment (coefficient of variation included in the legend and shown in Supplementary Figure S2), while Supplementary Figure S3 provides the data per biological replicate. Despite some intrinsic individual variability, the outcomes were consistent in independent biological replicates (Supplementary Figure S3A–D). The effect of MTAs nullified the ability of endothelial cells to respond to shear stress by morphological reorganization. Combined these findings strongly support the hypothesis that active remodeling of the MT network is essential for endothelial cell alignment and elongation responses to laminar shear stress.

### Flow-induced microtubule acetylation is necessary for EC elongation

**FIGURE 2:**
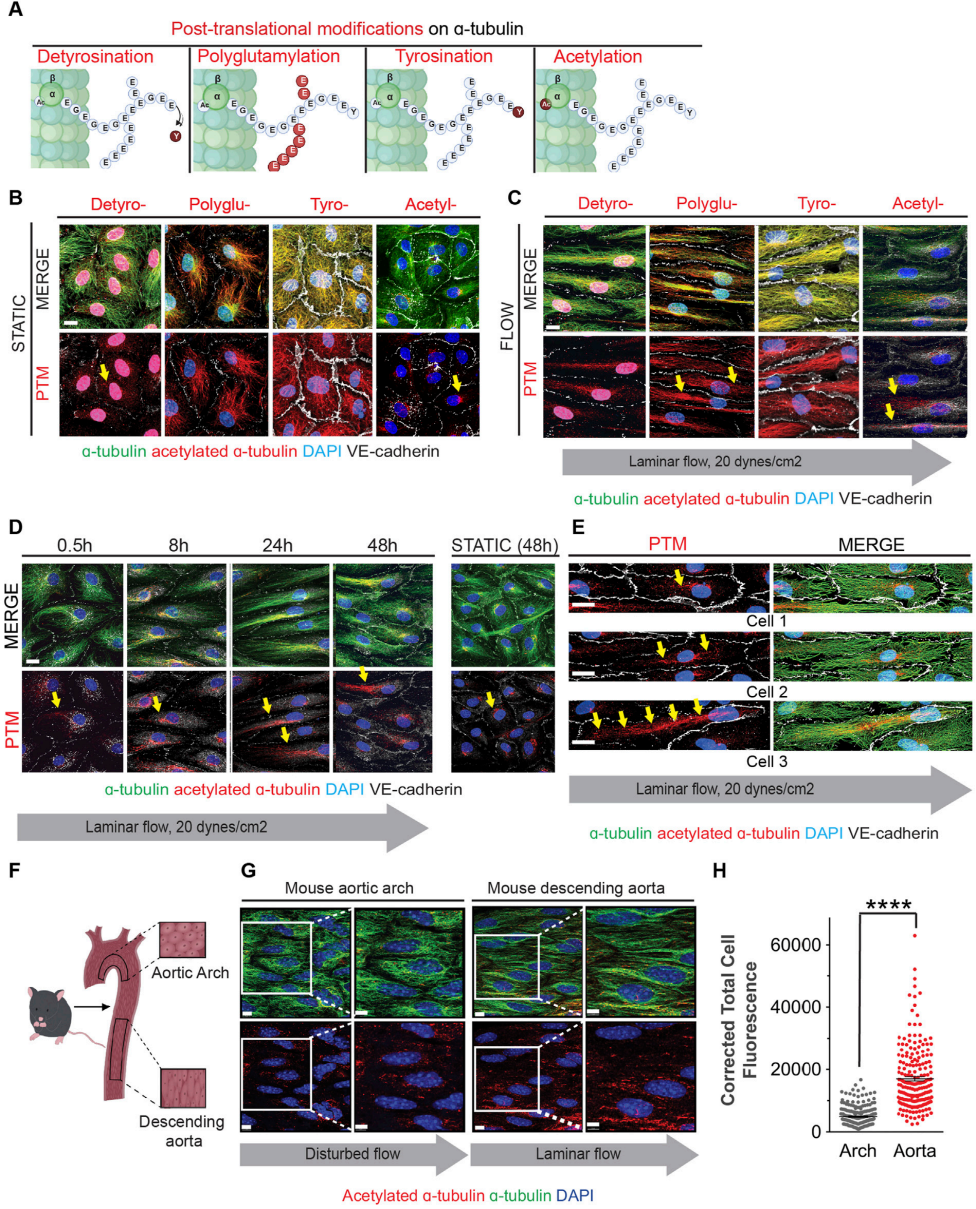
Fluid shear stress increases MT acetylation as endothelial cells undergo elongation and alignment in response to flow. **(A)** Schematic of post-translational modifications (PTMs) on the C-terminal end of α-tubulin (marked and labeled in red); detyrosination (detyro-), polyglutamylation (polyglu-), tyrosination (tyro-) and acetylation (acetyl-). **(B)** Immunofluorescence staining of post-translational modifications (red) and tubulin of HAECs exposed to static for 48 hours. Nuclei stained with DAPI. Scale bar: 20 um. **(C)** Immunofluorescence staining of post-translational modifications (red) and tubulin of HAECs exposed to laminar shear stress for 48 h. Nuclei stained with DAPI. Scale bar: 20 um. **(D)** Time course of acetylation in HAECs exposed to laminar flow at indicated time points. Yellow arrows show localization of acetylation. Scale bar: 20 um. **(E)** Staining of acetylated a-tubulin within three different endothelial cells exposed to flow. Yellow arrows point to localization of acetylation. **(F)** Schematic of the mouse aorta and arch. **(G)** Enface staining of the aortic arch and descending aorta with acetylated α-tubulin (red), α-tubulin (green), and for nuclei (DAPI). Scale bar: 10 um. **(H)** Quantification of acetylation of the aorta and arch. Data shown as mean ±SD. Mann-Whitney U test. ****p < 0.0001.

As MTs are subjected to several types of post-translational modifications, we next inquired whether some of these modifications were altered by exposure of endothelial cells to shear stress. Specifically, we assessed MT detyrosination, polyglutamination, tyrosination, and acetylation (Figure 2A). Initially we examined the distribution and relative abundance of these modifications by immunocytochemistry (Figure 2B). This first-pass evaluation indicated that MT acetylation was increased after exposure to shear stress and that its distribution was also altered over time (Figures 2B, C). In fact, MT acetylation under laminar shear stress expanded rapidly from a Golgi-centric pattern to an elongated pattern in which microtubules extended toward one or both cellular poles (Figures 2D, E). Importantly, while MT filled the entire cytoplasm, only a subset of MT in the center of the cell were acetylated (Figure 2E).

To ascertain whether acetylation of microtubules was associated with laminar shear stress *in vivo*, we evaluated endothelial cells from aortic arch regions exposed to either disturbed or laminar blood flow. The lesser curvature of the aortic arch experiences oscillatory and disturbed flow patterns associated with the rhythmic cycles of systole and diastole; in this region, ECs are polygonal and do not align in the direction of flow (Figures 2F, G). In contrast, ECs in the descending aorta are exposed to laminar shear stress and are both elongated and aligned in the direction of blood flow (Figure 2G). Furthermore, levels of acetylated alpha-tubulin were higher in the descending aorta when compared to the aortic arch (Figure 2H).

**FIGURE 3:**
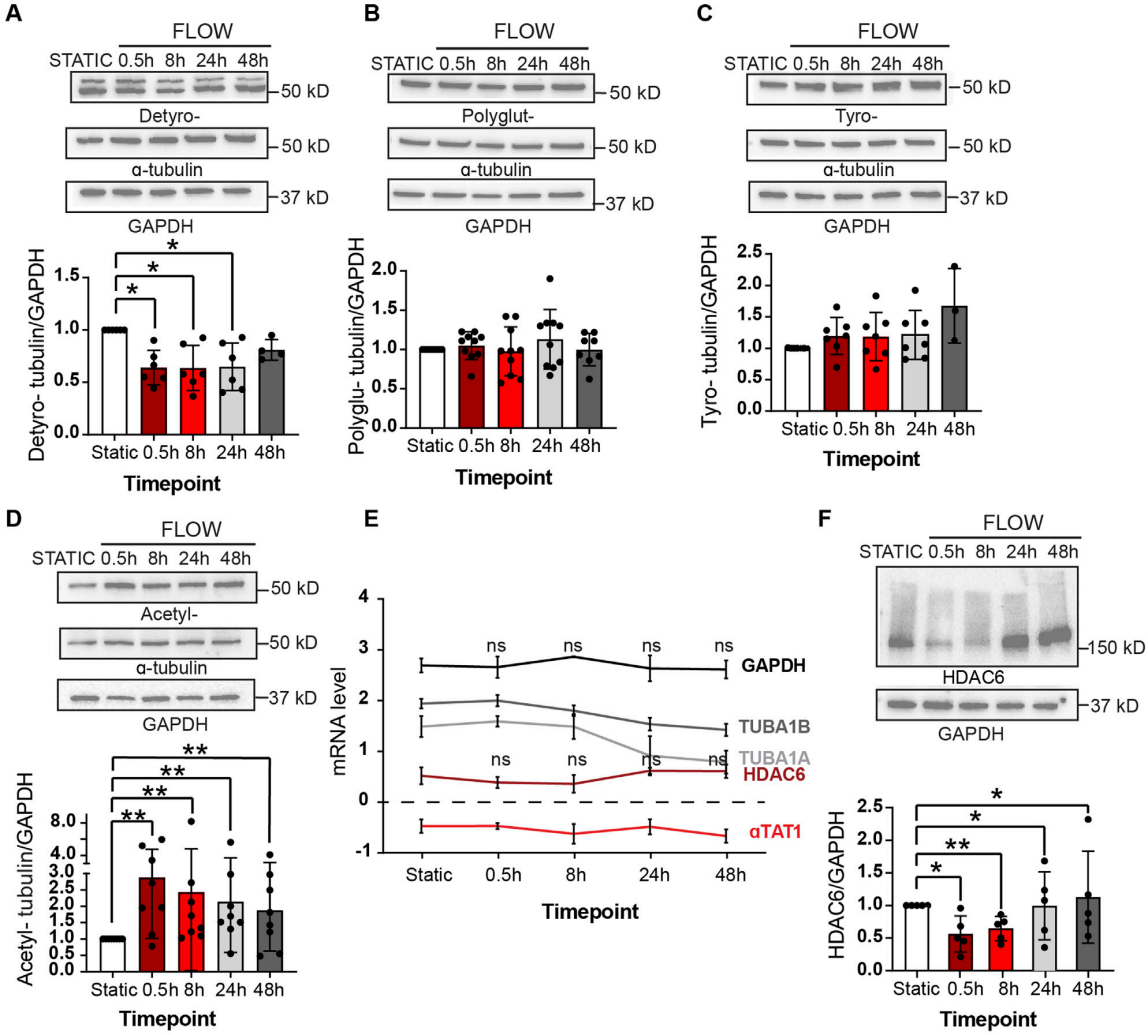
Shear stress alters post-translational modifications under shear stress Protein expression of PTMs at static (48 h), 0.5, 8, 24 and 48 h of shear stress. **(A)** Detyrosination **(B)** Polyglutamylation **(C)** Tyrosination **(D)** Acetylation. One sample Wilcoxon test. *n* = at least 3 technical replications with 3 biological replicates. **(E)** Transcriptomic data showing mRNA levels of various genes over 48 h of shear stress. Mann-Whitney U test. *n* = 4 biological replicates. **(F)** Protein expression of HDAC6 levels at indicated time course of static and laminar flow. Proteins were normalized to GAPDH. *n* = 5 technical replicates. One samplet-test. Data shown as mean ± SD. *p < 0.05, **p < 0.01.

A more expansive assessment of microtubule post-translational modifications was conducted by immunoblot analyses with multiple biological replicates (Figures 3A–D). Upon initiation of flow, we observed a statistically significant decrease in alpha-tubulin detyrosination which returned to static levels after 48 h (Figure 3A). No changes were noted in the levels of polyglutamination or tyrosination (Figures 3B, C). However, a rapid increase in acetylated alpha-tubulin was observed over the same time course when compared to static conditions. Significantly, alpha-tubulin experienced a 2.5 fold increase in acetylation over the first 30 min after initiation of flow (Figure 3D; Supplementary Figure S4 for uncropped individual blots).

The observed response of MT-acetylation to flow was fast and robust and we then explored the potential mechanism associated with such change. Steady-state acetylation of proteins is reached by the net effect of acetylases and deacetylases. In the case of microtubules, this is accomplished by the acetylase alpha TAT1 and the deacetylase HDAC6 (Kalebic et al., 2013; Haggarty et al., 2003; Hubbert et al., 2002; You et al., 2020; North et al., 2007). One potential explanation to the increase in MT acetylation would be either the transcriptional increase of alpha TAT1 or the decrease of HDAC6 by flow. However, we found that both transcripts were fairly stable upon flow induction (Figure 3E). In contrast, levels of HDAC6 protein declined precipitously upon exposure of endothelial cultures to flow conditions (Figure 2F). This reduction of HDAC6 protein explains the significant accumulation of MT acetylation at early time points.

Next, we explored the biological implications of pharmacological blockade of acetylases and deacetylases to endothelial cell elongation and alignment under flow conditions. Tubacin is a highly selective and reversible HDAC6 inhibitor that permeates the cell membrane and leads to rapid increase in tubulin acetylation (Hideshima et al., 2016; Itoh et al., 2007). In contrast, 5z-7-oxozeanol, an inhibitor of TAK1 prevents TAT-1 mediated acetylation of microtubules (Shah et al., 2018). Prior to exposing cells to tubacin and 5z-7-oxozeaenol, we titrated the levels of these inhibitors to reach changes in acetylation while maintaining cellular function and MT dynamics. For this, we again used EB3-GFP to monitor MT activity (Supplementary Figure S1E, Video S2) while also assessing efficient modifications on MT acetylation.

**FIGURE 4:**
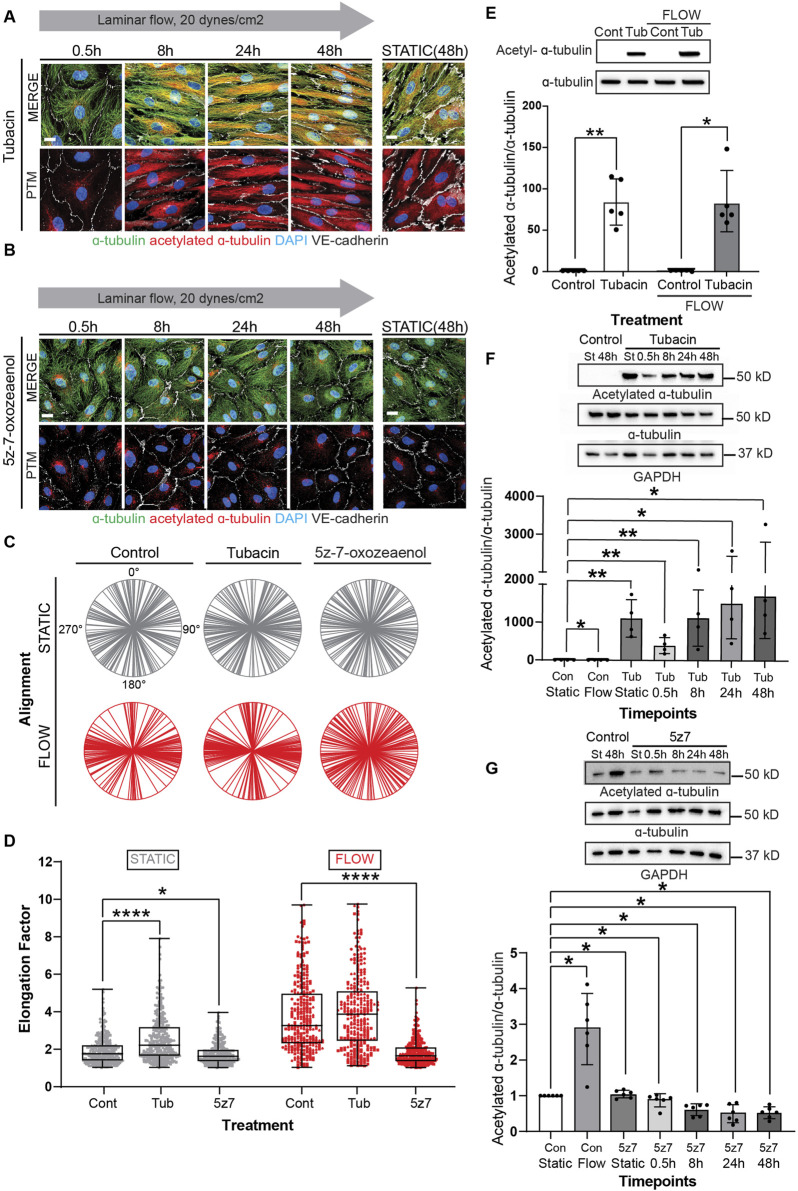
Modulation of MT acetylation by Tubacin and 5z-7-oxozeaenol impairs EC alignment and elongation under shear stress. **(A, B)** Immunofluorescence images of a time course of endothelial cells treated with Tubacin or 5z-7-oxozeaenol, respectively. Cells were stained for α-tubulin (green), acetylated α-tubulin (red), nuclei (DAPI) and VE-cadherin (white). Scale bar: 20 um. **(C)** Radial graphs. *n* = 100 cells per condition, 3 biological replicates. Coefficients of variations for static (left to right) 0.53, 0.5, 0.48 and flow (left to right) are 0.17, 0.13 and 0.58 (Supplementary Figure S2). **(D)** Elongation Factor. *n* = 300 cells per condition, 3 biological replicates. Kruskal-Wallis Test with Dunn’s correction. **(E)** Protein expression of acetylated α-tubulin and α-tubulin comparing control and Tubacin-treated cells with and without flow. *n* =5 technical replicates with 3 biological replicates. One sample Wilcoxon test. **(F)** Representative blot and quantification of a time course of endothelial cells treated with Tubacin with control cells exposed to static and laminar flow. *n* = 4 technical replicates. Unpaired t-test. **(G)** Protein quantification of a time course of endothelial cells treated with 5z-7-oxozeaenol with control cells exposed to static and laminar flow for 48 hours. *n* = 6 technical replicates. One sample Wilcoxon test. Data shown as mean ± SD. *p < 0.05, **p < 0.01, ***p < 0.001, ****p < 0.0001.

Treatment of confluent endothelial cultures with tubacin resulted in abundant acetylation of MT with accelerated elongation (Figure 4A). In contrast, blockade of MT acetylation with 5z-7-oxozeaenol prevented endothelial cell elongation in response to laminar shear stress (Figure 4B). Evaluation of cell alignment in confluent cultures showed that tubacin enhanced parallel organization of cells in direction to flow, while 5z-7-oxozeaenol impaired this collective response to flow (Figure 4C). The alignment of individual biological replicates under each treatment is shown in Supplementary Figure S5A–D. Importantly, quantification of endothelial elongation revealed that even under static conditions, tubacin promoted cell elongation, an effect that was more pronounced under flow conditions (Figure 4D). Increased MT acetylation by tubacin was confirmed by immunoblots revealing an increase of approximately 80-fold over control-treated cultures (Figure 4E; Supplementary Figure S6 for uncropped blots). The effect of tubacin on microtubule acetylation is gradual but extremely robust and noted as early as 30 min by immunoblot (Figure 4F). Importantly, flow-mediated induction in acetylation was significantly blocked by 5z-7-oxozeaenol (Figure 4G). Interestingly, base-line acetylation was retained in the presence of the TAK1 inhibitor (Figures 4B, G) implying that 5z-7-oxozeaenol only blocks the acetylation that is induced by flow. Note that for these experiments, treatment with 5z-7-oxozeaenol was done concurrently with application of flow, but even after 48h, base-line acetylation was retained.

### Maintenance of elongation requires MT remodeling despite prior increases in acetylation

**FIGURE 5:**
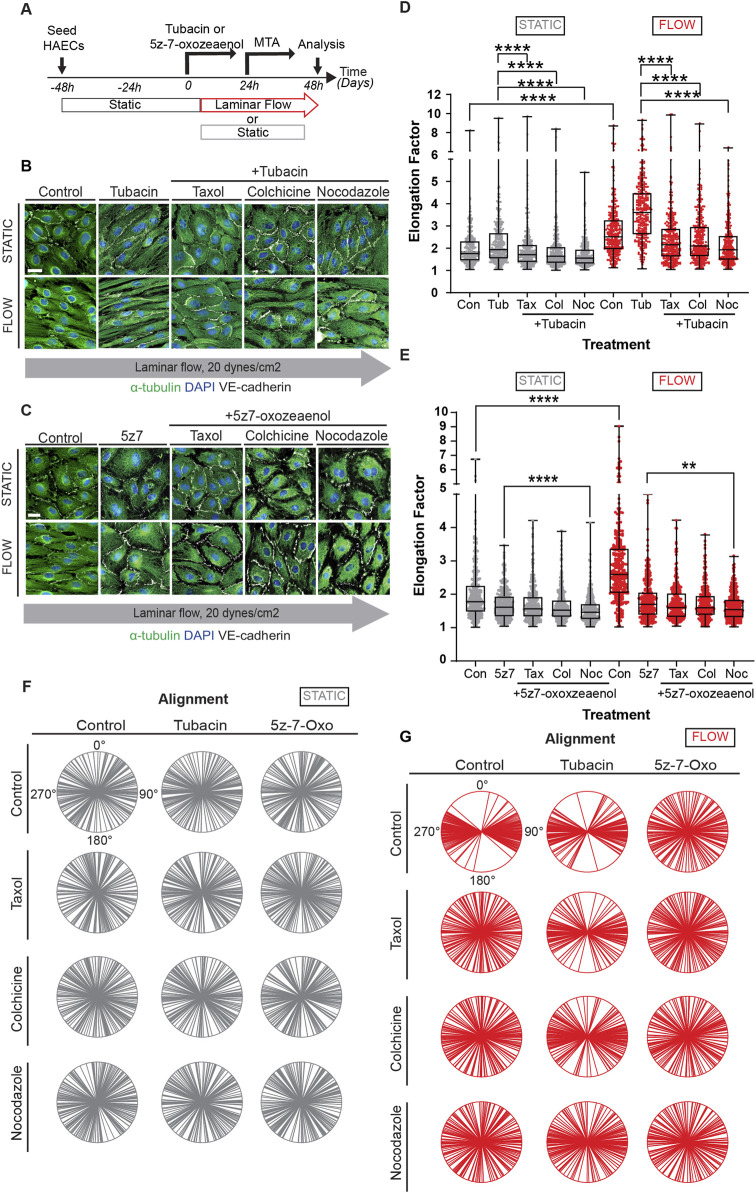
Constant microtubule dynamics and controlled acetylation is required for proper EC responses to flow. **(A)** HAECs were pre-treated with pharmacological inhibitors, Tubacin and 5z-7-oxozeaenol, and then treated with MTAs (taxol, nocodazole, or colchicine) under static or flow. Immunofluorescence images of cells sequentially treated with **(B)** Tubacin **(C)** 5z-7-oxozeaenol and then with MTAs under static or shear stress conditions. Images show staining for α-tubulin (green), nuclei (DAPI) and VE-cadherin (white). Scale bar: 20um. (D-E) Analysis of elongation factor of (B) and **(C)**. Data are shown as mean ± SD; *n* = 300 cells per condition, 3 biological replicates. Kruskal-Wallis Test with Dunn’s correction. **(F, G)** Radial graphs of **(B)** and **(C)**. Coefficients of variations of **(F)** for static (column by column) are 0.57, 0.58, 0.58, 0.52, 0.63, 0.55, 0.61, 0.57, 0.59, 0.58, 0.62 and 0.59. Coefficients of variations of **(G)** for flow (column by column) are 0.28, 0.53, 0.48, 0.54, 0.28, 0.36, 0.54, 0.52, and 0.5, 0.52, 0.57 and 0.56 (Supplementary Figure S2). *n* = 100 cells per condition, 3 biological replicates. **p < 0.01, ***p < 0.001, ****p < 0.0001.

Acetylation of microtubules is thought to increase their stability. Interestingly, we found that levels of MT acetylation were drastically increased when endothelial cells were exposed to taxol (Supplementary Figure S7), indicating that status of MT dynamics feedbacks on acetylation. Along these lines, it is unclear if shear-induced elongation of endothelial cells is a stable phenotype or if microtubule remodeling is continuously necessary to maintain this phenotype. Thus, we performed experiments in which cells were exposed to flow for 24 h, in the presence or absence tubacin or 5z-7-oxozeaenol, followed by an additional 24 h of laminar flow with MT disrupting agents (Figure 5A). Evaluation of such cultures by immunocytochemistry revealed that cell elongation was reversed by MTA drugs even when in the presence of tubacin (Figure 5B), while no change was observed when similar experiments were done with 5z-7oxozeaenol (Figure 5C). Quantification of cell elongation in the presence of tubacin under static conditions further showed a statistically significant difference between tubacin alone and tubacin in the presence of MTAs (Figure 5D). Under flow conditions, cells lost their elongated phenotype and became polygonal when exposed to inhibitors of MT dynamics even in the context of shear stress (Figure 5D). When exposed to 5z-7oxozeaenol in the presence of MTAs, a statistically significant difference was only observed when in the presence of nocodazole (Figure 5E). Despite these changes, intercellular contacts were retained under all conditions (Figures 5B, C).

Alignment was also evaluated in these experiments. As expected, no changes in alignment were observed under static conditions (Figure 5F and individual biological replicates in Supplementary Figure S8). Cultures under laminar shear stress and treated with tubacin tended to show a different trend for alignment than for elongation when exposed to inhibitors of MT dynamics (Figure 5G). In fact, cultures exposed to tubacin were more resistant to becoming randomized in their alignment when exposed to taxol and colchicine than cultures not exposed to tubacin (Figure 5G). This effect was more pronounced in some biological replicates than in others (Supplementary Figure S8). The findings are consistent with the notion that acetylation increases MT stability making them more resistant to depolymerization. In addition, these results underline the constant requirement of MT dynamics to mechanotransduction.

### HDAC6 and alpha-TAT1 regulate MT acetylation during EC responses to laminar shear stress

**FIGURE 6:**
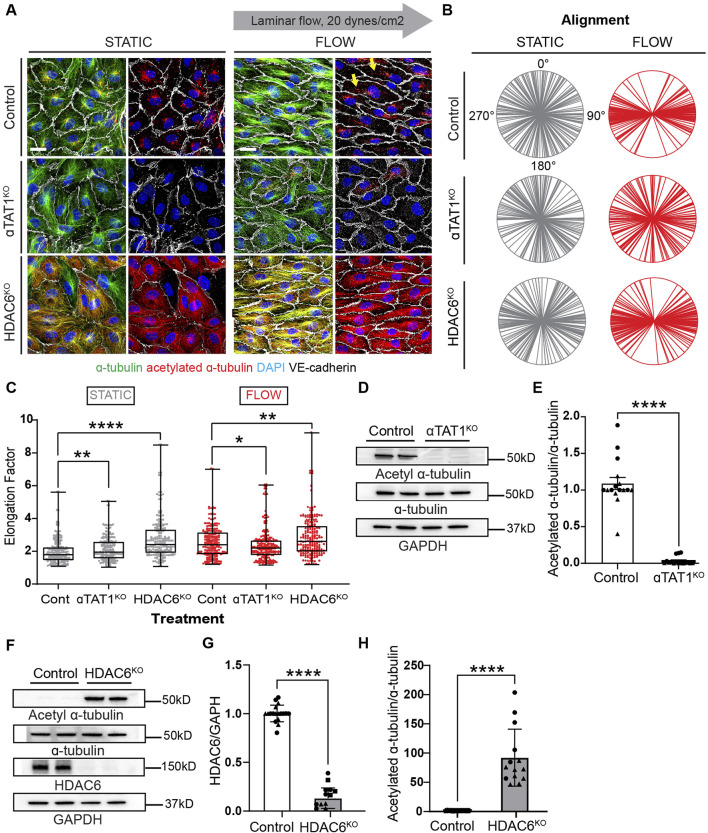
Genetic inactivation of HDAC6 and αTAT1 leads to altered acetylation levels and disruptions of cell shape under shear stress. **(A)** Immunofluorescence images of acetylation and total tubulin in control, HDAC6KO, and αTAT1KO human umbilical vein endothelial cells (HUVECs) under static and flow. Scale bar: 20 um. Yellow arrows point to the increased acetylation seen in control ECs under shear stress. **(B)** Radial graphs comparing control vs KO HUVECs under static and shear stress. *n* = 100 per condition, 2 biological replicates. Coefficients of variations for static (first column) are 0.57, 0.52, 0.56 and for flow (second column) are 0.23, 0.53 and 0.5 (Supplementary Figure S2). **(C)** Elongation factor comparing control and KO HUVECs. *n* = 300 per condition, 2 biological replicates. Mann-Whitney U test. **(D)** Protein expression of α-tubulin, acetylated α-tubulin and GAPDH comparing control and αTAT1KO HUVECs. **(E)** Quantification of acetylation between control and aTAT1KO HUVEC. *n* = 16 technical replicates, 3 biological replicates. One sample Wilcoxon test. **(F)** Immunoblots of HDAC6, α-tubulin, acetylated α-tubulin and GAPDH comparing control and HDAC6KO HUVECs. **(G)** Protein expression of HDAC6 between control and HDAC6KO; *n* = 18 technical replicates, 3 biological replicates. One sample Wilcoxon test. **(H)** Protein expression of acetylated α-tubulin from control compared to HDAC6KO HUVECs. *n* = 14 technical replicates, 3 biological replicates. One sample Wilcoxon test. Data shown as mean ± SD, except **(D)** which is shown at mean ± SEM. *p<0.05 **p < 0.01, ***p < 0.0001.

To mitigate the potential off-target effects of pharmacological inhibitors (tubacin and 5z-7oxozeaenol) on the emerging conclusions, we performed experiments where HDAC6 and alpha-TAT1 were inactivated by CRIPR on endothelial cells. It has been well-accepted that HDAC6 is the enzyme that removes acetyl groups from tubulin (Haggarty et al., 2003), while alpha-TAT1 can acetylate tubulin on lysine 40 (Kalebic et al., 2013). Although these two enzymes are not the only ones that can have this function on MT. We indeed found that CRISPR-mediated inactivation of alpha TAT1 results in a very efficient blockade of MT-acetylation (Figure 6A). Furthermore, elongation and alignment were inhibited when this enzyme was absent in endothelial cells (Figures 6A–C). In contrast, inactivation of HDAC6 resulted in robust microtubule acetylation, promoted endothelial cell elongation and alignment (Figures 6A–C; Supplementary Figure S9A–C for individual biological replicates). Similarly to tubacin exposure, inactivation of HDAC6 resulted in a statistically significant increase in endothelial cell elongation even under static conditions (Figure 6C). Biochemical evaluation of the changes in microtubule acetylation was also conducted using immunoblots. Inactivation of alpha alpha TAT1 blocked microtubule acetylation (Figures 6D, E; Supplementary Figures S9D–H for uncropped blots). In contrast, the absence of HDAC6 resulted in a robust increase (nearly 100-fold) in microtubule acetylation (Figures 6F–H). The impressive elevation of acetylation in HDAC6 null cultures underlines the essential impact of this enzyme in controlling steady-state levels of MT acetylation.

## DISCUSSION

**FIGURE 7:**
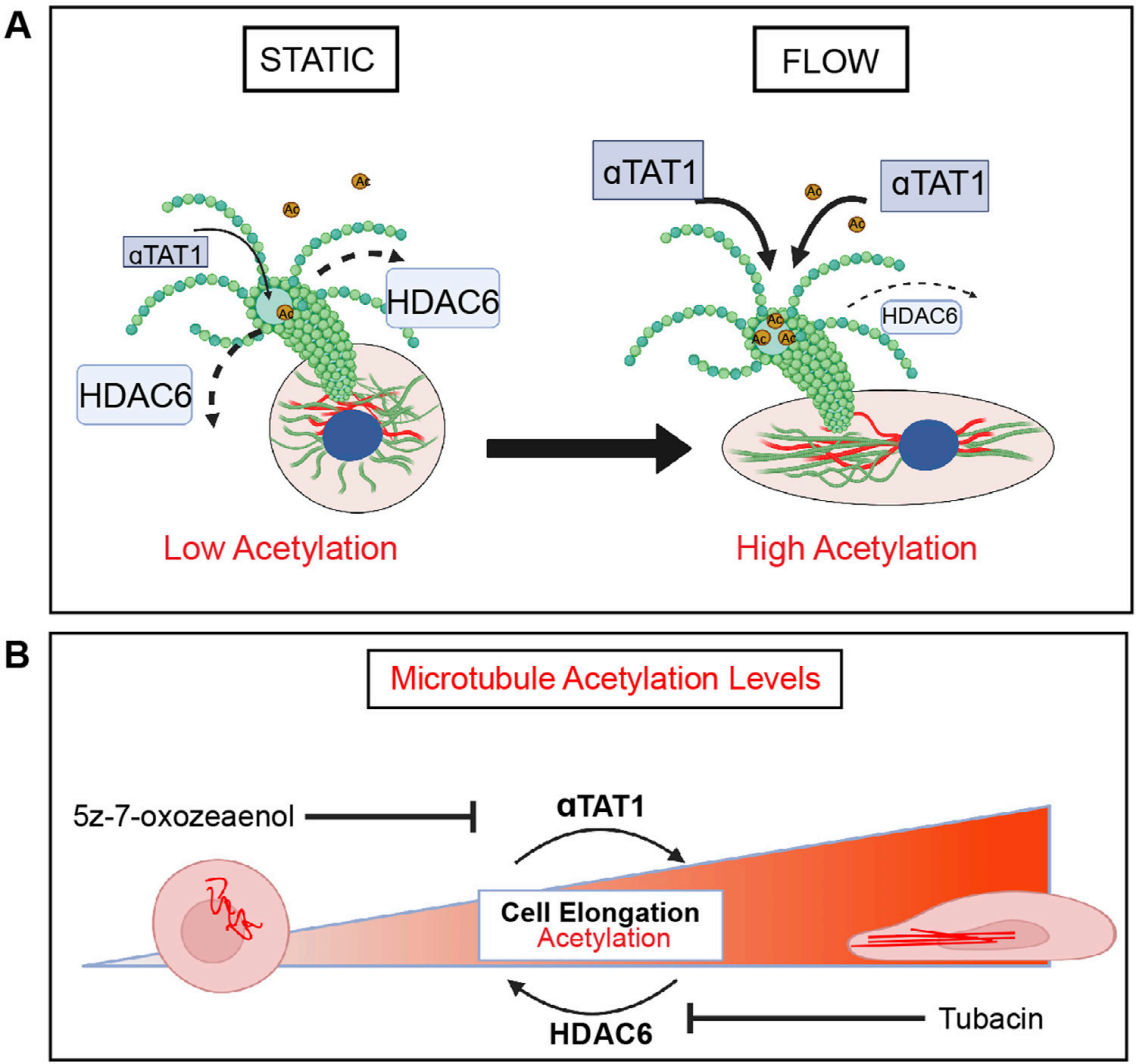
HDAC6 and aTAT1 are key mediators of EC mechanotransduction through regulation of microtubule acetylation. **(A)** In response to shear stress, endothelial cells elongate and align in the direction of flow through remodeling of the cytoskeleton which includes reorganization of microtubules. There is a flow-dependent increase in acetylation that corresponds to endothelial cell elongation and alignment. HDAC6 and αTAT1 regulate the timing (kinetics) and localization of acetylation on α-tubulin in response to shear stress in endothelial cells. HDAC6 is found at its lowest at the onset of flow. **(B)** Disruption of HDAC6 and αTAT1, the key regulators of MT acetylation by pharmacological inhibition or through genetic inactivation results in impairment of endothelial cell mechanotransduction. Loss of HDAC6 leads to significant acetylation and faster kinetics in elongation. Inactivation of αTAT1 leads to significant reduction in acetylation with reduced elongation.

Using a combination of pharmacological inhibitors and genetic manipulations, this study demonstrates the essential requirement of MT dynamics on endothelial cell mechanotransduction in response to laminar shear stress. Specifically, endothelial cell elongation and alignment were blocked in the presence of agents that either inhibit polymerization or depolymerization of MT. We also showed that acetylation of microtubules increases rapidly when endothelial cells are exposed to laminar shear stress *in vitro* and *in vivo* (Figure 7A). Furthermore, genetic or pharmacological prevention of this post-translational modification hinders cell elongation and alignment in response to laminar shear stress (Figure 7B). Together, these findings highlight the contributions of microtubules to the most basic responses of endothelial cells to fluid flow.

The cytoskeleton is a common denominator in a growing array of cellular responses to mechanical force. All three major components of the cytoskeleton have been implicated in responses to physical force, including actin, intermediate filaments, and microtubules (Chien, 2007; Campinho et al., 2020). Thus, it is not surprising that endothelial responses to shear stress have been linked with an expanding number of alterations in these proteins and an expanding appreciation for the role of cytoskeletal regulators in their highly varied physiologic contexts. In fact, remodeling of the endothelial actin cytoskeleton and focal adhesions have been described in detail (Helmke et al., 2003; Noria et al., 2004; Mott and Helmke, 2007; Gegenfurtner et al., 2018). Intermediate filaments, and in particular vimentin has been implicated in the transmission of forces sensed by PECAM in response to shear stress (Collins et al., 2012). Conspicuously absent from this framework was the role of microtubules relatively few studies reporting microtubule network elongation in response to shear stress (Zielinski et al., 2018). Importantly, previous investigators recognized an increase in MT acetylation in response to shear stress, although those studies did not address the kinetics, deacetylases and acetylases involved, mechanisms, or effects on alignment (McCue et al., 2006).

Unexpectedly and in broad strokes, the contribution of the three major cytoskeletal proteins in sensing and responding to shear stress appears to be largely not-overlapping. For example, genomic inactivation of vimentin, the main and highly abundant intermediate filament in endothelial cells has no impact on endothelial cell elongation in response to flow (Salvador et al., 2022). This is not to negate other roles of vimentin in mechanotransduction. Nonetheless it is clear that cell elongation in response to flow is not part of its portfolio of vimentins activities. In contrast, the actin cytoskeleton owns a unique position in the regulation of EC polarity, cell-cell and cell-matrix through its interactions with focal adhesions and cell adhesion molecules, like cadherin complexes (Wojciak- Stohard and Ridley, 2003; Noria et al., 2004) both in the presence and absence of flow. Furthermore, the actin cytoskeleton is critical at counterbalancing excessive intracellular tension through cross talk with phosphoinositide (PI) 3-kinase (Thodeti et at., 2009). At present, microtubules stand alone in regulating cell shape changes in response to shear stress, a function that couples well with its roles in intracellular transport. While not tested here, it is possible that microtubule acetylation, known to alter affinity for motor proteins could contribute to elongation by playing a formative role in delivering key components needed for polarized membrane expansion and resorption. Along these lines, it is interesting that actin displays interactions specifically with acetylated microtubules, through Arp2/3 regulation of cargo trafficking (Yoshimura et al., 2021).

We also found that the MT network adapts differently in regions of the arterial tree responding to different types of flow stress. ECs on the inner curvature of the aortic arch are exposed to disturbed blood flow, are polygonal in morphology, and exhibit lower levels of acetylated MTs. In contrast, ECs found in the descending aorta are exposed to unidirectional (laminar) blood flow. ECs in descending aorta are elongated in the direction of flow and express a MT network that is directionally organized and displaying increased levels of acetylated-alpha-tubulin in the region of the cell facing into the flow, upstream of the nucleus. A potential function of increased acetylation of alpha-tubulin could be the acquisition of additional resilience to mechanical strain, helping to buttress cellular responses to shear stress. Along these lines, it is interesting that acetylation of microtubules is known to augment their stability, enhance flexibility and resistance against repeated mechanical stresses (Xu et al., 2017; Prokop et al., 2022; Saunders et al., 2022). An unexpected finding from our studies was that treatment with taxol alone, resulted in a remarkable increase in MT acetylation. Interestingly despite this high acetylation (similar to levels seen with tubacin), presence of taxol blocked endothelial cells from responding flow. The findings imply that microtubule dynamics are continuously required for endothelial responses to flow, as high acetylation cannot override the need for microtubule remodeling. Importantly, acetylation of alpha-tubulin protects long-lived MTs from mechanical aging by making them less susceptible to stress-induced damage while preventing pre-existing MT lattice defects from spreading (Portran et al., 2017). Whereas accumulation of stress-induced damage was observed in deacetylated MTs subjected to continuous stress (Portran et al., 2017). Cilia are structures highly enriched for acetylated tubulin, however in agreement with previous literature (Dinsmore and Reiter, 2016) we found that only a low percentage of cells under laminar shear stress had cilia.

The potential links between MT acetylation and TGF-b signaling in the context of shear stress did not escape us. As demonstrated here, alpha-TAT1 is the critical acetyltransferase responsible for microtubule acetylation in response to shear stress. Interestingly, TGF-beta activated kinase (TAK1) phosphorylates alpha-TAT1 at Ser237 promoting its activation and, in turn, MT acetylation (Shah et al., 2018; Zhu et al., 2021). These molecular and functional relationships link TGF-beta signaling to MT acetylation and endothelial cell elongation. In fact, the use of 5z-7 oxozeaenol, an inhibitor of TAK1, clearly shows the relevance of this kinase in microtubule acetylation in response to flow. Interestingly, 5z-7 oxozeaenol blocked flow-induced MT acetylation; however, base-line acetylation of MT in proximity to the Golgi was retained, as noted by staining and immunoblotting. The findings imply that base-line acetylation in might be regulated by a distinct subset of acetylases. It is peculiar that a clear consequence in endothelial cells that lack endoglin or Smad4 is the loss of endothelial cell elongation in response to shear stress (Poduri et al., 2017; Sugden et al., 2017). The implication is that inadequate MTacetylation in response to flow might impact endothelial cell shape and perhaps contribute to the development of hereditary hemorrhagic telangiectasia in patients with mutations in those genes.

In summary, our data expands our current understanding of how endothelial cells respond to shear stress and bring microtubule acetylation at the forefront of cell shape remodeling in the context of physical forces.

LIMITATIONS: There are several limitations of the study, particularly in relation to the specificity of acetylation and possible confounding effects of pharmacological and genetic inactivation on other targets. In fact, HDAC6 deacetylates multiple other cytoplasmic targets, some of these highly relevant to endothelial cell biology such as cortactin. The same can be stated for alpha-TAT1. Alternative approaches would be to mutate the lysine associated with the acetylation. Unfortunately, this is also problematic in the case of microtubules, as lysine 40 is also a site for methylation. Thus, mutating this site will also be confounded by how absence of methylation impacts MT function in the context of shear stress. Despite these limitations, the combination of pharmacological and genetic inhibition of HDAC6 does bring to light the constant activity of this enzyme on endothelial MT and its regulation is likely to be the rate-limiting step in the maintenance and distribution of acetylated microtubules. Future studies evaluating HDAC6 regulation and control, as well as the impact of MT acetylation in transport of cargo are likely important next steps to fully understand endothelial polarity in response to shear stress.

## Data Availability

The raw data presented in the study are deposited in the BioImage Archive, accession number S-BIAD1345 (https://www.ebi.ac.uk/biostudies/BioImages/studies/S-BIAD1345). The code used for elongation and alignment analyses are deposited in Github (https://github.com/GoyalLab/EndothelialCellAnalysis).
