# Supplementary material for: Endothelial cell elongation and alignment in response to shear stress requires acetylation of microtubules
Source: Front Physiol. 2024 Sep 10;15:1425620. doi: 10.3389/fphys.2024.1425620 (PMC11420013; doi:10.3389/fphys.2024.1425620)
Supplement: Supplementary file 6 [file DataSheet1.pdf]

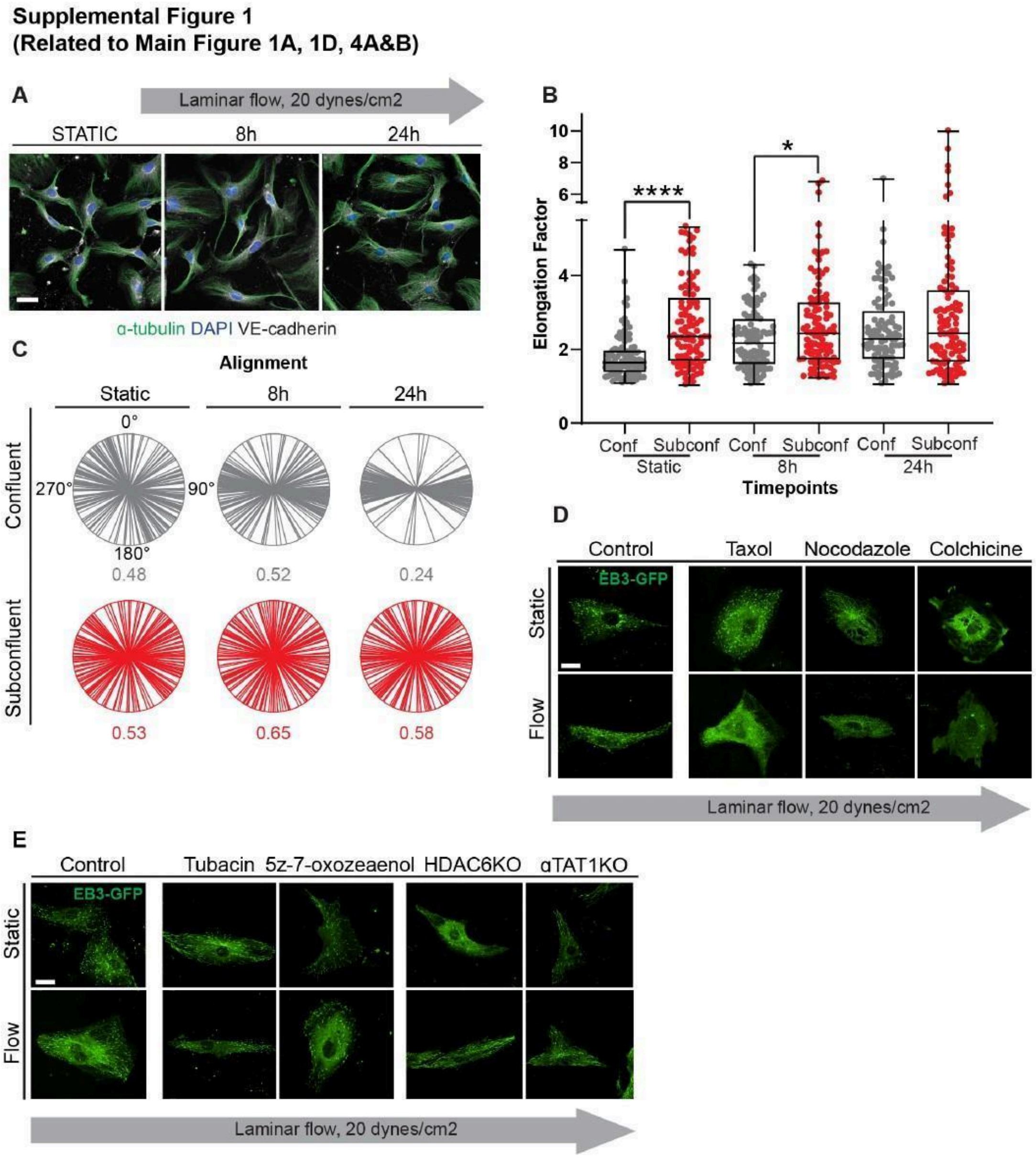

FIGURE S1| Supplementary Figure 1. (Related to Main Figure 1A, 1D) (A) Immunofluorescent images of sub-confluent human aortic endothelial cells (HAEC) under static, 8h, and 24h of shear stress. Scale bar: 90um. **(B)** Elongation factor comparing confluent and sub-confluent endothelial cells. Mann Whitney U test. *n*= 100 cells per condition**. (C)** Radial graphs comparing the alignments between confluent and sub-confluent ECs with indicated coefficient of variation values. **(D)** Static images of the videos provided for EB3-GFP transfected HUVECs treated with MTAs under static and laminar shear stress conditions. Scale bar: 20um. **(E)** Static images from the videos provided from non-transduced and KO cell lines transfected with EB3-GFP. Similarly, EB3-transfected ECs were treated with Tubacin or 5z-7-oxozeaenol to view polarity dynamics. Scale bar: 20um.

Supplemental Figure 2
(Related to Main Figure 1,4,5,6)

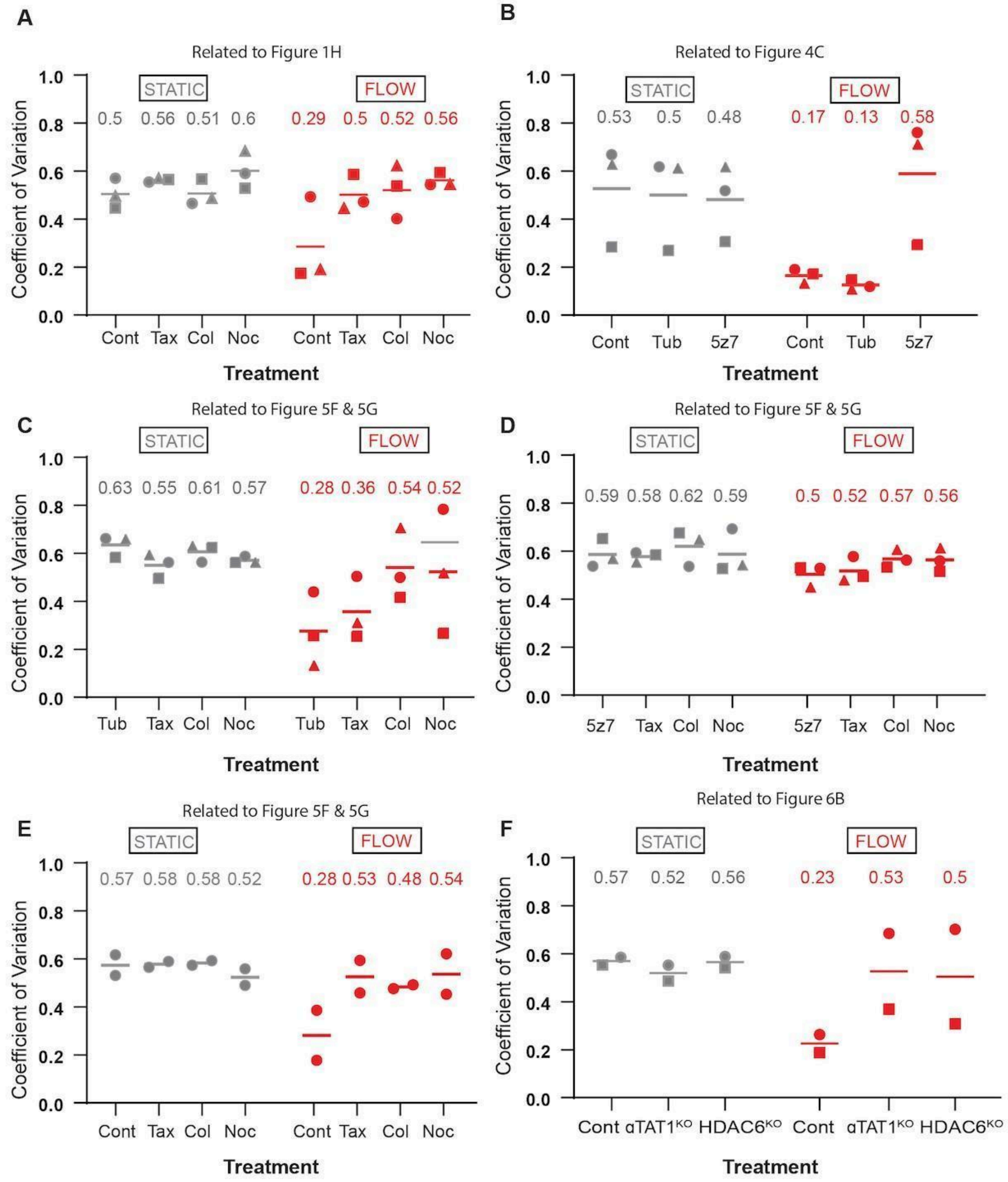

Supplementary Figure 2. (Related to Figure 1,4,5,6). Coefficient of Variation analysis of radial graphs related to Figures 1 **(A**), 4 **(B)**, 5 **(C-E)**, 6 **(F)**. Each graph represents the variability of analyzed cells of each condition in the data set. Each point represents *n***=** 100 cells except Figure 4 which is *n* = 40 or 100 cells. Data shown as mean.

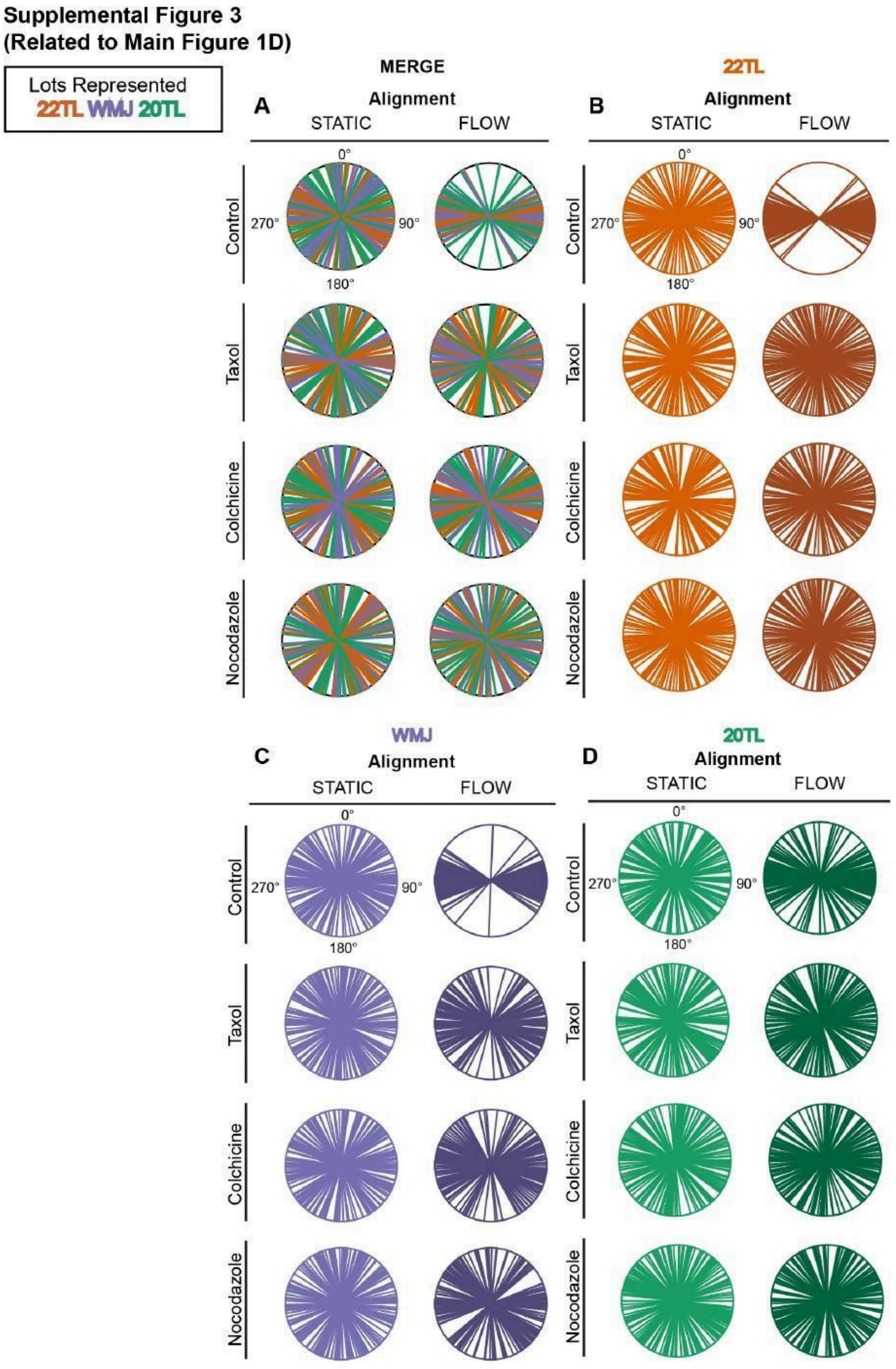

Supplemental Figure 3. (Related to Main Figure 1D) (A) Merged radial graphs of HAECs treated with MTAs in represented colors for each biological replicate **(B)** 22TL (orange), **(C)** WMJ (lavender), **(D)** 20TL (green) in the presence and absence of shear stress. Merged plots: *n*= 33, 33, 34 cells per condition. Individual plots: *n*= 40, 100, 100 per condition respectively.

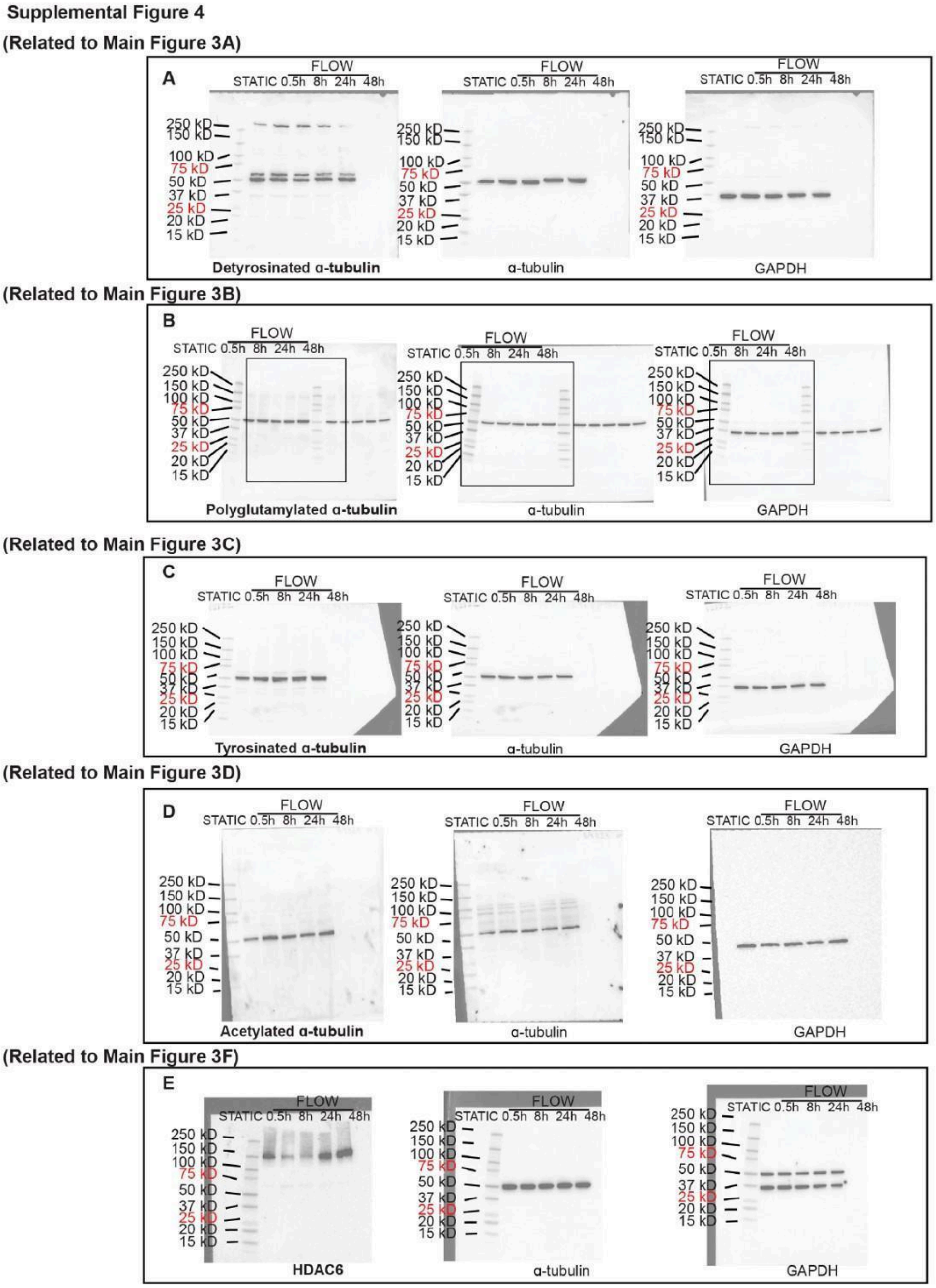

Supplementary Figure 4. (Related to Figure 3). (A-D) Original, uncropped blots that evaluates changes in post-translational modifications (detyrosination, polyglutamylation, tyrosination, and acetylation) over a time course of 48 hours under laminar shear stress with housekeeping genes, α-tubulin and GAPDH.

Supplemental Figure 5
(Related to Main Figure 4C)

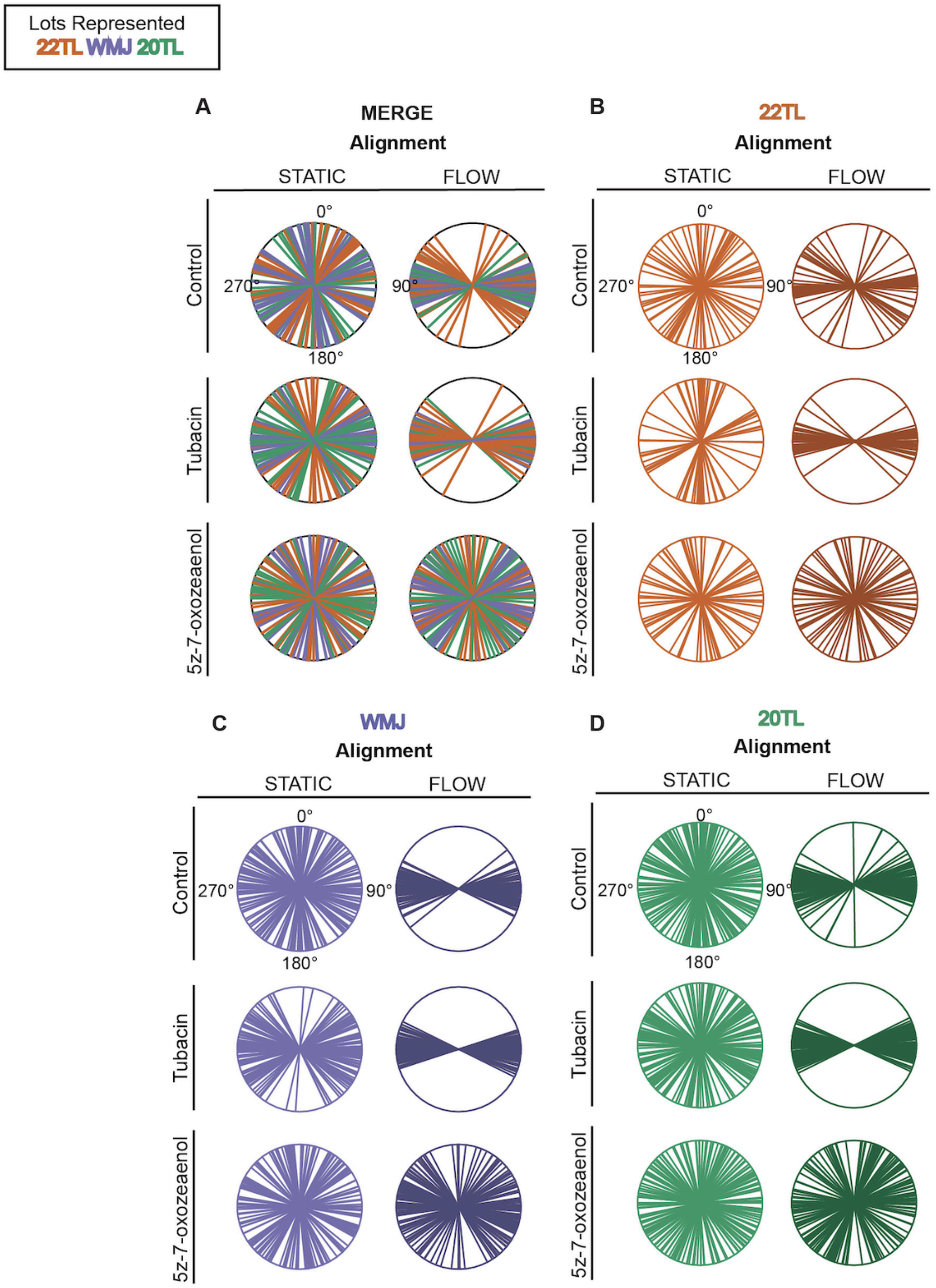

Figure 5. (Related to Figure 4C) (A) Merged radial graphs of HAECs treated with DMSO (control), Tubacin or 5z-7-oxozeaenol in represented colors for each biological replicate **(B)** 22TL (orange), **(C)** WMJ (lavender), **(D)** 20TL (green) in the presence (Flow) and absence (Static) of laminar shear stress. Merged plots: *n*= 33, 33, 34 cells per condition. Individual plots: *n*= 40, 100, 100 per condition respectively.

Supplemental Figure 6

(Related to Figure 4E)

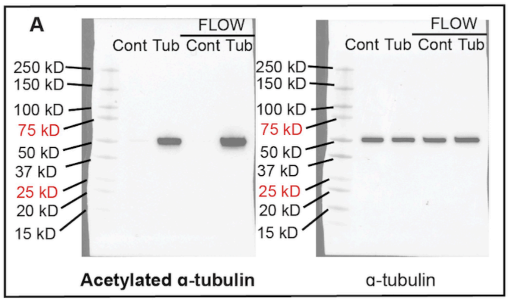

(Related to Figure 4F)

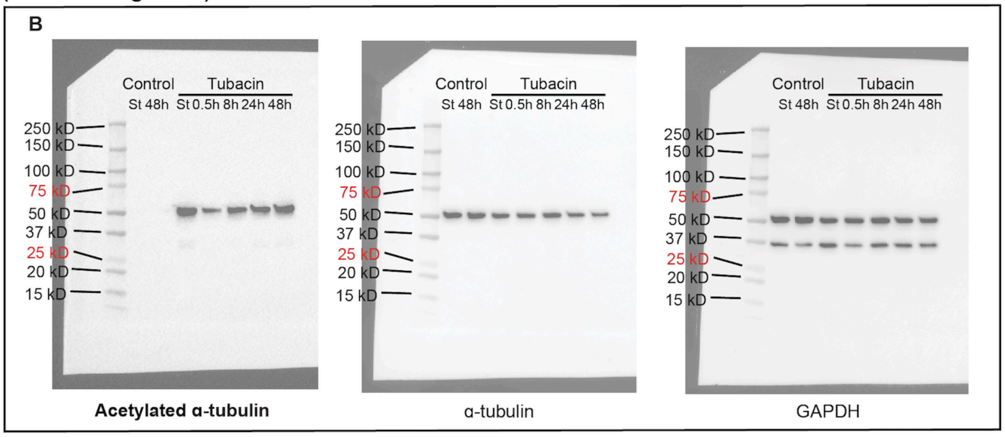

(Related to Figure 4G)

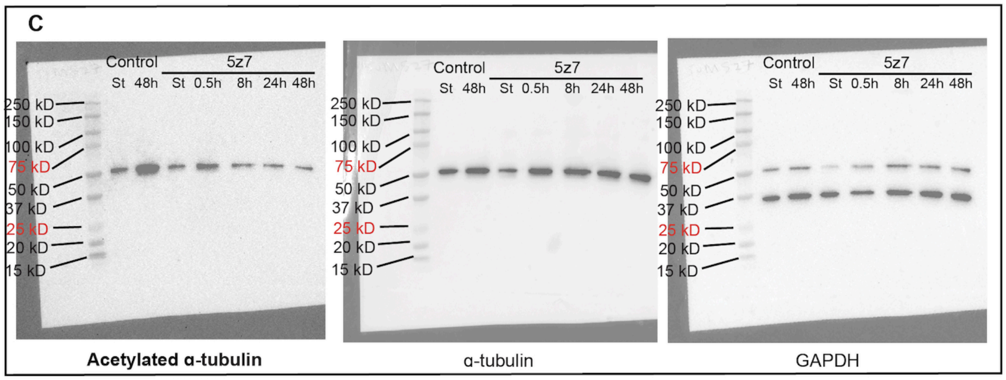

Supplementary Figure 6. (Related to Main Figure 4E-G). (A) Original, uncropped blots of HAECs treated with Tubacin compared to control cells. **(B)** Original, uncropped blots of Control and Tubacin-treated cells under a 48h time course. **(C)** Original, uncropped blots of Control and 5z-7-oxozeaenol -treated cells under a 48h time course.

Supplemental Figure 7

(Related to Main Figure 1D)

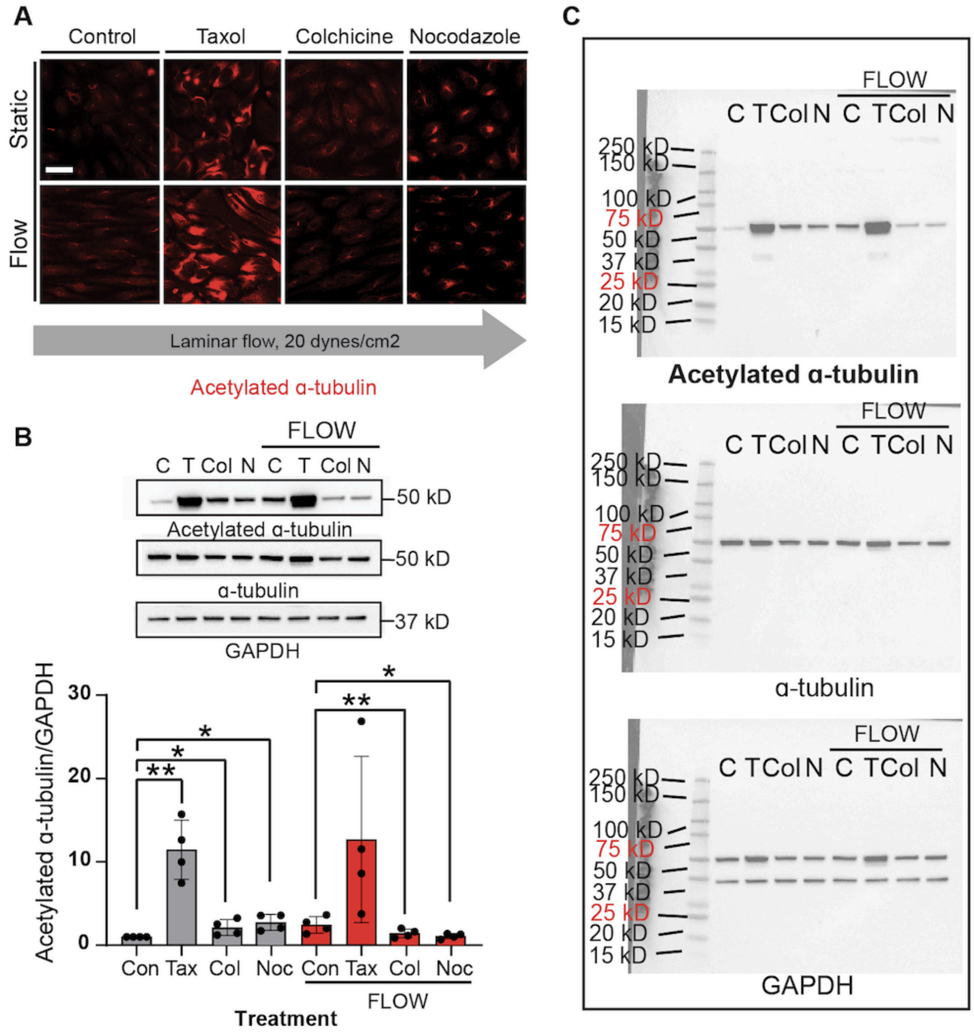

Supplemental Figure 7. (A) Immunofluorescence staining of acetylated α-tubulin expression of ECs treated with MTAs. Scale bar: 20um. **(B)** Protein expression of acetylated αtubulin comparing control and microtubule targeting agents (MTA)-treated ECs under static and flow. One sample t-test. *n*= 4. **(C)** Original, uncropped blots of protein expression of acetylation of ECs treated with MTAs with housekeeping genes, a-tubulin and GAPDH.

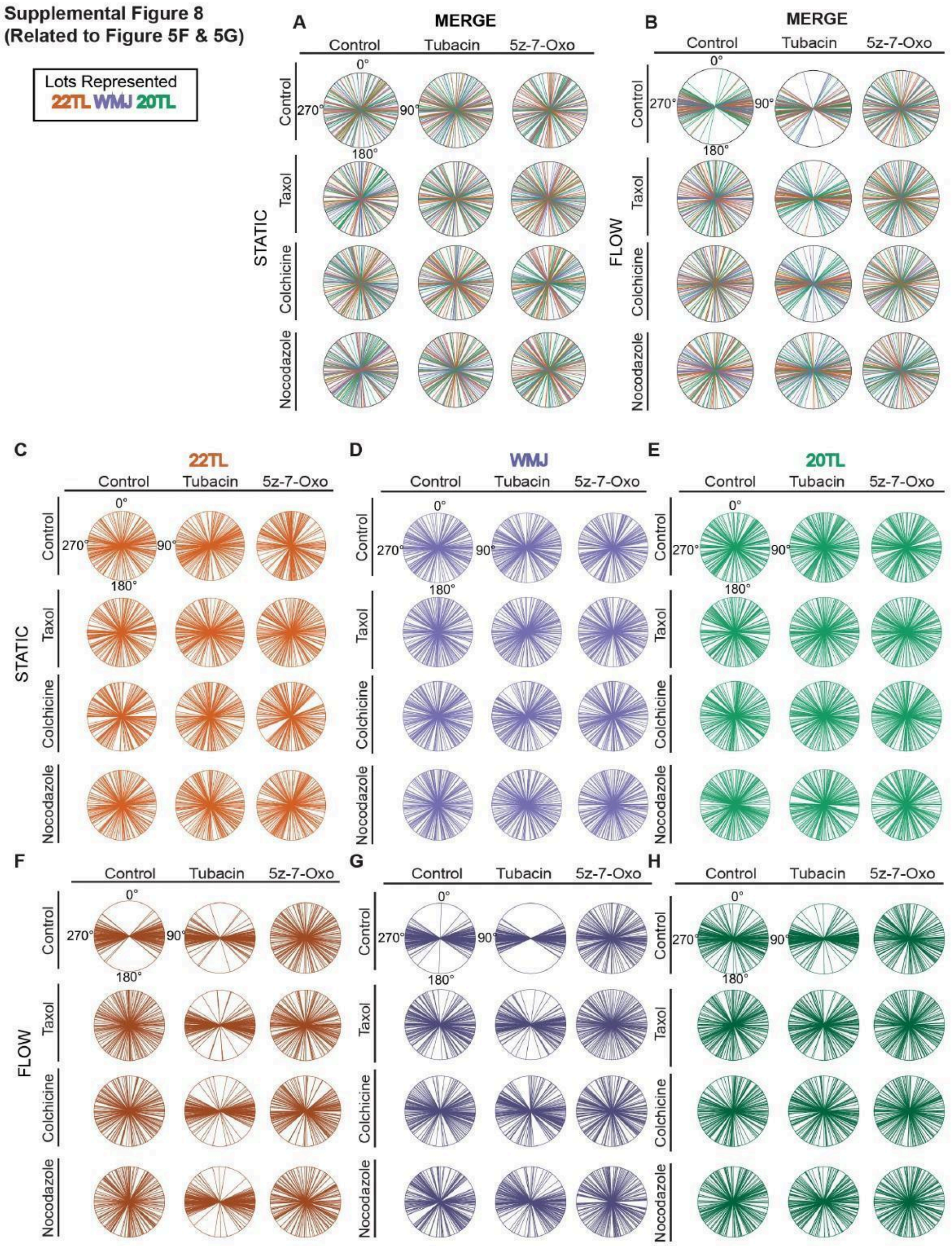

Supplementary Figure 8. (Related to Main Figure 5) (A, B) Merged radial graph of HAECs treated MT dynamics inhibitors and Tubacin or 5z-7-oxozeaenol in represented colors for each biological replicate **(C, F)** 22TL (orange), (**D, G)** WMJ (lavender), **(E, H)** 20TL (green) under static and shear stress conditions. Merged plots: *n*= 33, 33, 34 cells per condition. Individual plots: *n*= 40, 100, 100, cells per condition respectively.

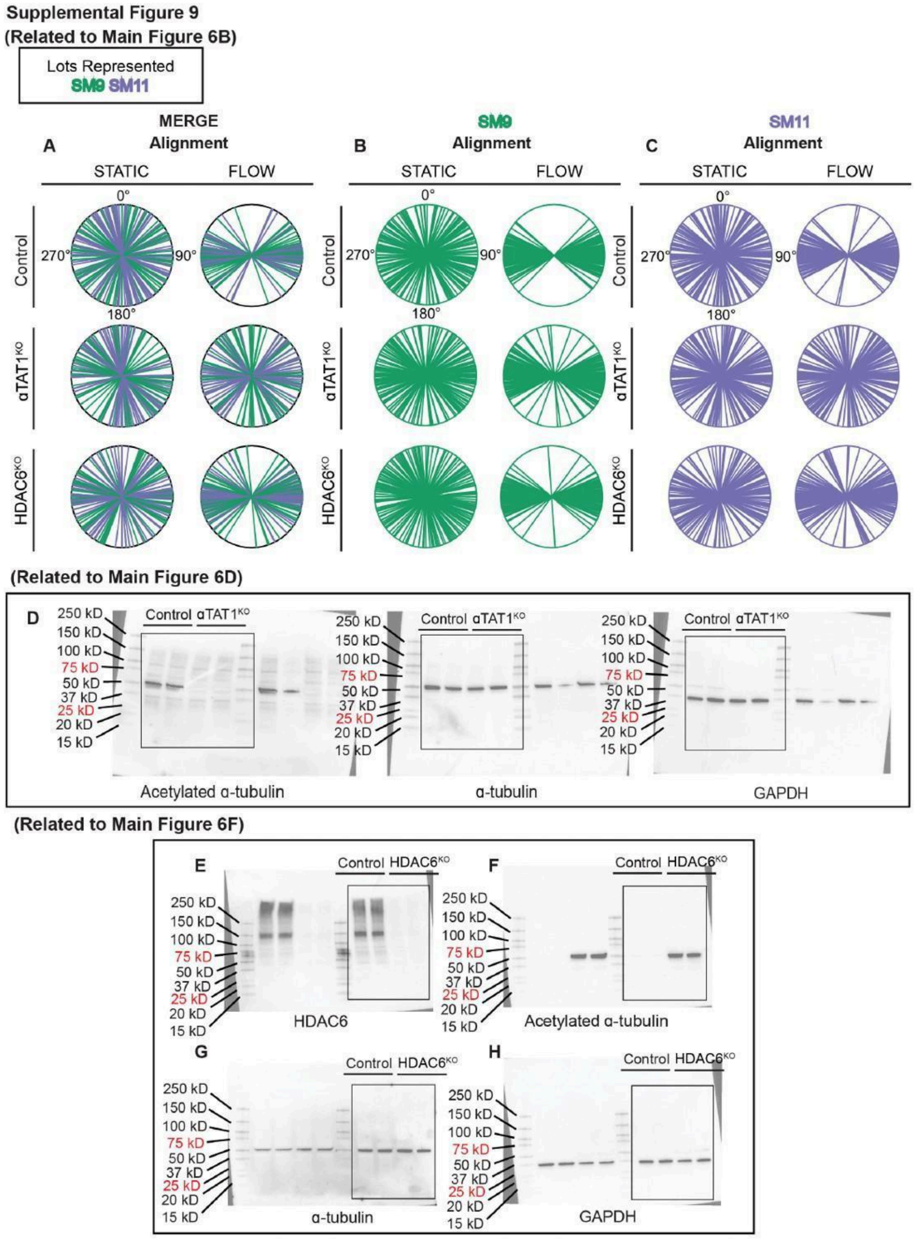

Supplementary Figure 9 (Related to Main Figure 6) (A) Merged radial graph of HUVEC knocked down for HDAC6 or aTAT1 or non-transduced. For each biological replicate **(B)** SM9 (green), and **(C)** SM11 (lavender) under static and shear stress. Merged plots: *n*=50 cells per condition. Individual plots: *n*= 100 cells per condition. **(D)** Original, uncropped blots comparing control and aTAT1KO HUVECs with knockdown expression of acetylation a-tubulin in aTAT1KO cells. **(E)** Original, uncropped blots comparing control and HDAC6KO HUVECs. Blot results confirm the **(F)** knockdown of HDAC6 with **(F)** elevated acetylation levels in HDAC6KO cells (**G, H**) with housekeeping genes.

Supplementary Video 1 (Related to Suppl S1D) Live-imaging videos of endothelial cells transfected with EB3-GFP. Use of EB3-GFP shows the polarity of microtubules and is a marker of microtubule growth. **(A)** Top Row: Transfected endothelial cells were treated with (left to right) DMSO, taxol, nocodazole and colchicine. Bottom Row: Endothelial cells were similarly treated with MTAs and exposed to laminar shear stress for 48-hours. Control cells show functional microtubule dynamics, with intact presence of EB3-GFP comets and continuous marks along microtubules. Cells treated with taxol show shorter movement of EB3 and nocodazole cells show pulsatile reduced trajectory along microtubules. Colchicine cells show complete disruption of EB3-GFP and no movement of the comets are seen.

Supplementary Video 2 (Related to Suppl S1E) Live-imaging videos of endothelial cells transfected with EB3-GFP with chemical or genetic perturbation of acetylation of microtubules **(A)** Top Row: Transfected endothelial cells were treated with (left to right) DMSO, Tubacin, 5z-7-oxozeaenol and knocked down for HDAC6 and aTAT1. Bottom Row: Endothelial cells were similarly treated and subjected to laminar shear stress for 48-hours. Control cells show function microtubule dynamics with EB3 comets running along microtubules. There are similar phenotypes in the movement of EB3-GFP seen across the treatments regardless of suppression or overexpression of MT acetylation.
